# Synthesis of spiro[isoindole-1,5’-isoxazolidin]-3(2*H*)-ones as potential inhibitors of the MDM2-p53 interaction

**DOI:** 10.3762/bjoc.12.278

**Published:** 2016-12-20

**Authors:** Salvatore V Giofrè, Santa Cirmi, Raffaella Mancuso, Francesco Nicolò, Giuseppe Lanza, Laura Legnani, Agata Campisi, Maria A Chiacchio, Michele Navarra, Bartolo Gabriele, Roberto Romeo

**Affiliations:** 1 Dipartimento di Scienze Chimiche, Biologiche, Farmaceutiche e Ambientali, Via S.S. Annunziata, 98168 Messina, Italy; 2 Dipartimento di Chimica e Tecnologie Chimiche, Università della Calabria, Via P. Bucci, 12/C, 87036 Arcavacata di Rende (CS), Italy; 3 Dipartimento di Scienze Chimiche, Biologiche, Farmaceutiche e Ambientali, Università di Messina, Viale F. Stagno d'Alcontres 31, 98166 Messina, Italy; 4 Dipartimento di Scienze del Farmaco, Università di Catania, Viale A. Doria, 95100 Catania, Italy; 5 Dipartimento di Chimica, Università di Pavia, Via Taramelli 12, 27100 Pavia, Italy

**Keywords:** antitumor agents, DFT studies, 1,3-dipolar cycloaddition, docking studies, spiro-compounds

## Abstract

A series of spiro[isoindole-1,5-isoxazolidin]-3(2*H*)-ones has been synthesized by 1,3-dipolar cycloaddition of *N*-benzylnitrone with isoindolin-3-methylene-1-ones. The regio- and stereoselectivity of the process have been rationalized by computational methods. The obtained compounds show cytotoxic properties and antiproliferative activity in the range of 9–22 μM. Biological tests suggest that the antitumor activity could be linked to the inhibition of the protein–protein p53-MDM2 interaction. Docking measurements support the biological data.

## Introduction

The p53 tumor suppressor protein is a transcriptional factor that plays a key role in the regulation of several cellular processes, including apoptosis, DNA repair, and angiogenesis [1–4]. The murine double minute 2 (MDM2) protein is the primary cellular inhibitor of p53, functioning through direct interaction with p53 [5]: tumoral cells show an overexpression of MDM2 which suppresses the functions of the p53 protein [5–8].

**Figure 1:**
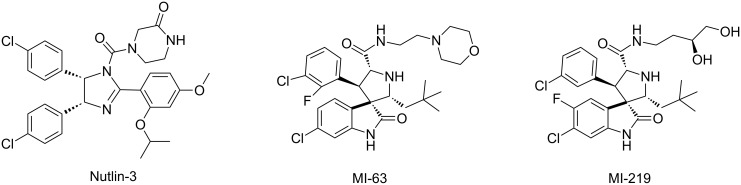
Some relevant MDM2-p53 interaction inhibitors.

The design of non-peptide, small-molecule inhibitors that block the MDM2-p53 interaction has been sought as an attractive strategy to activate p53 for the treatment of cancer and other human diseases [9–11]. Major advances have been made in the design of lipophilic small-molecule inhibitors of the MDM2-p53 interaction in recent years, and several compounds have moved into advanced preclinical development or clinical trials [12–14]. Potent MDM2-p53 inhibitors, such as Nutlin-3 [12] and the spirooxindoles, for example MI-63 and MI-219, [15–18] have demonstrated cellular activity consistent with inhibition of MDM2-p53 binding and have shown in vivo antitumor activity [15,19] (Figure 1).

Isoindolinones, belonging to the alkaloids family, are found in many natural products such as vitedoamine A, chilenine, lennoxamine, magallanesine and nuevamine [20–25]. These compounds possess a lot of pharmacological activities such as anxiolytic/anticonvulsant, TNFα-inhibitory, antiangiogenic, 5-HT antagonistic/antidepressant [26–30], PARP-1-inhibitory [31], histone deacetylase inhibitory [32] and cytotoxic activity.

Recently, MDM2-p53 inhibitors based on an isoindolinone scaffold [33–34] have been reported. These latter results demonstrate the versatility of the isoindolinone scaffold as MDM2-p53 inhibitor and show that significant improvements in potency may be gained by modest structural modifications.

Introducing structural diversity into the isoindolinone scaffold can represent an important approach towards the design of new chemotherapeutics.

**Figure 2:**
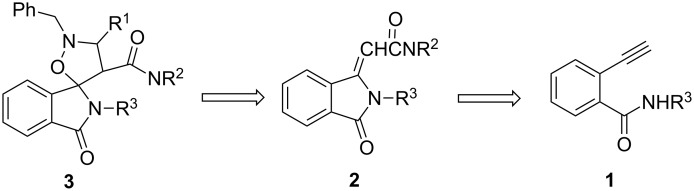
Retrosynthetic route to spiro[isoindole-1,5-isoxazolidin]-3(2*H*)-ones **3**.

On the basis of these considerations, we have designed a route towards a new class of potential MDM2-p53 inhibitors **3**, through the construction of the spiro[isoxazolidin-isoindolinone] system. The synthetic scheme (Figure 2) exploits the strategy of the 1,3-dipolar cycloaddition of nitrones on the substrate isoindolin-3-methylene-1-one **2**, obtained by a recent methodology of a PdI_2_ catalyzed aminocarbonylation-*N*-heterocyclization of 2-ethynylbenzamides **1** [35].

The rationale of our choice is based on molecular docking data. Using the published structure of the MDM2–p53 binding site, we have employed computational methods and focused library synthesis based on the isoindolinone template, to develop compounds with inhibitory activity. These studies have resulted in the identification of a number of potential MDM2-p53 interaction inhibitors. Biological tests confirm our initial hypothesis indicating for compounds **3** an antiproliferative activity in the range of 9–22 μM: the antitumor activity appears to be linked to the inhibition of the protein–protein p53-MDM2 interaction.

## Results and Discussion

### Chemistry

**Scheme 1:**
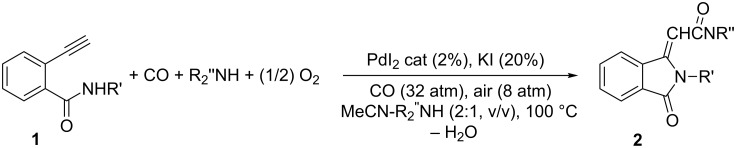
Synthesis of compounds **2**.

The synthetic scheme towards the construction of the spiro[isoxazolidin-isoindolinone] system **3** starts from isoindolinones **2** which have been synthesized, as reported [35], by Pd-catalyzed aminocarbonylation-*N*-heterocyclization of 2-ethynylbenzamides **1**. The latter are easily accessible through ethynylation of *N*-substituted 2-iodobenzamides, with secondary amines (Scheme 1).

**Scheme 2:**
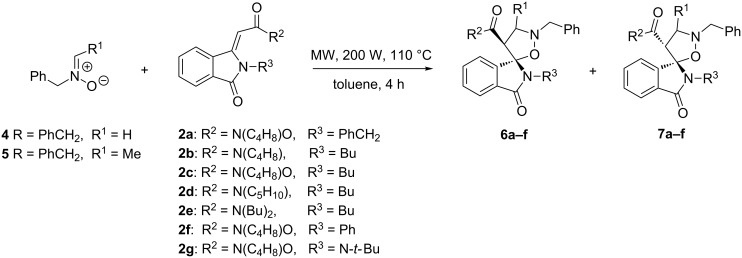
Synthesis of **6a–f** by 1,3-dipolar cycloaddition.

Compounds **2a–e**, as *E/Z* mixtures, have been reacted with nitrone **4**. Different experimental conditions have been exploited: by reacting the nitrone and dipolarophile in toluene solution under reflux, the formation of products was not observed, even after extension of the reaction time to 72 h. Under these conditions the process led only to decomposition of the nitrone with the recovery of unaltered isoindolinone. The best results have been obtained by performing the 1,3-dipolar cycloaddition in toluene at 110 °C for 4 h, under microwave irradiation: cycloadducts **6a–e** have been isolated in 38–60% yield, as major isomers, together with isomers **7** as minor adducts and unreacted isoindolinones **2a–e** mainly in *E* configuration (Scheme 2, Table 1).

**Table 1:** Synthesis of **6a–f** by 1,3-dipolar cycloaddition.

Entry	Nitrones	Dipolarofile	(*Z/E* ratio)	Product	Ratio	Yield %^a^

1	**4**	**2a** R^2^ = N(C_4_H_8_)O, R^3^ = PhCH_2_	2.2	**6a**/**7a**	85:15	60
2	**4**	**2b** R^2^ = N(C_4_H_8_), R^3^ = Bu	1.8	**6b**/**7b**^b^	95:5	55
3	**4**	**2c** R^2^ = N(C_4_H_8_)O, R^3^ = Bu	2	**6c**/**7c**^b^	94:6	40
4	**4**	**2d** R^2^ = N(C_5_H_10_), R^3^ = Bu	1	**6d**/**7d**^b^	99:1	35
5	**4**	**2e** R^2^ = N(Bu)_2_, R^3^ = Bu	1	**6e**/**7e**^b^	99:1	38
6	**4**	**2f** R^2^ = N(C_4_H_8_)O, R^3^ = Ph	only *Z* isomer	**6f**/**7f**	100:0	65
7	**4**	**2g** R^2^ = N(C_4_H_8_)O, R^3^ = N-*t-*Bu	only *E* isomer	**6g**/**7g**	0:100	10
8	**5**	**2a** R^2^ = N(C_4_H_8_)O, R^3^ = PhCH_2_	2.2	–	–	–

^a^Isomeric mixture; ^b^not isolated.

The cycloaddition reaction of **2f**, obtained only as *Z* isomer in the aminocarbonylation procedure, produces only **6f** in 65 % yield, while the reaction performed with **2g**, which is present only as *E* isomer, leads to adduct **7g** even if in low yield (10%). These set of experiments indicate that the *Z* isomers are more reactive than *E* derivatives and that the *Z* compounds give only cycloadducts **6**, while *E* lead only to adducts **7**.

The ^1^H NMR spectrum of the crude reaction mixture shows the stereoisomers **6a–f** as the main products, while stereoisomers **7a–f** are present as minor components or only in traces.

The cycloaddition reaction showed complete regioselectivity and a high stereoselectivity in favour of the [1(*RS*),4’(*RS*)]2,2’-dibenzyl-4’-substituted spiro[isoindole-1,5’-isoxazolidin]-3(2*H*)-ones **6a–f**.

The structure of adducts **6** and **7** has been elucidated by ^1^H NMR and ^13^C NMR spectroscopies and MS spectrometry. In particular, the ^1^H NMR spectrum of **6a**, chosen as model compound, shows the diagnostic resonance of the H9 proton at 3.83 ppm, while the methylene protons at C8 resonate at 3.71 and 3.41 ppm. In compound **7a**, H9 proton resonates at 2.70 ppm, while the methylene protons at C8 resonate at 3.84 and 4.08 ppm. The detailed long-range coupling analysis observed in the ^1^H,^13^C-HBMC confirms the attributions; thus, the long-range coupling between H9 and C7 (66.34 ppm) of the benzyl substituent at the nitrogen atom of the isoxazolidine ring, observed in compound **6a**, is in agreement with the proposed structure. Conversely, for **7a**, a long-range coupling was detected between H9 and C18 (43.40 ppm), the carbon atom of the benzyl group at N2.

**Figure 3:**
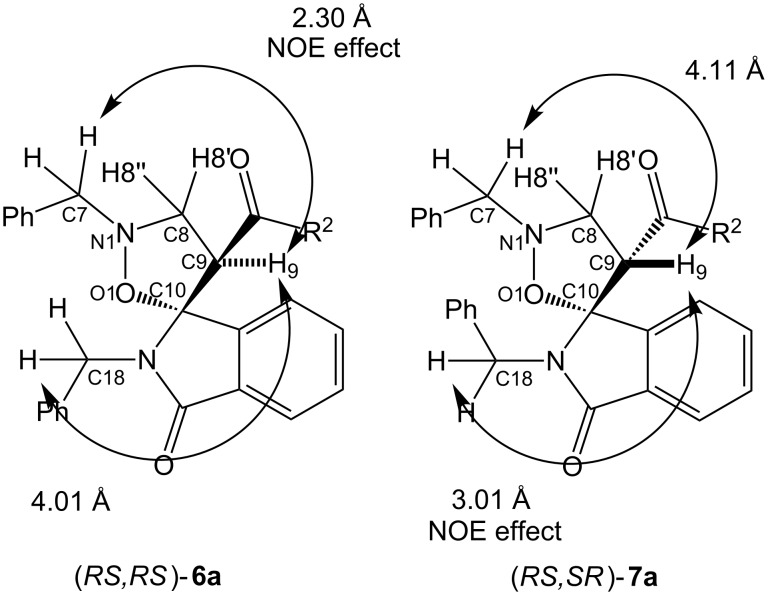
Selected NOESY observed for compounds **6a** and **7a**.

NOE experiments support the assigned stereochemical relationships. In agreement with the internuclear distance values, obtained from computational data (see Supporting Information File 1), irradiation of H9, in compound **6a**, induces a positive NOE effect for methylene protons at C7 (4.25 and 4.06 ppm), while, for **7a**, a NOE enhancement was observed for methylene protons at C18 (5.06 and 4.82 ppm) (Figure 3).

**Figure 4:**
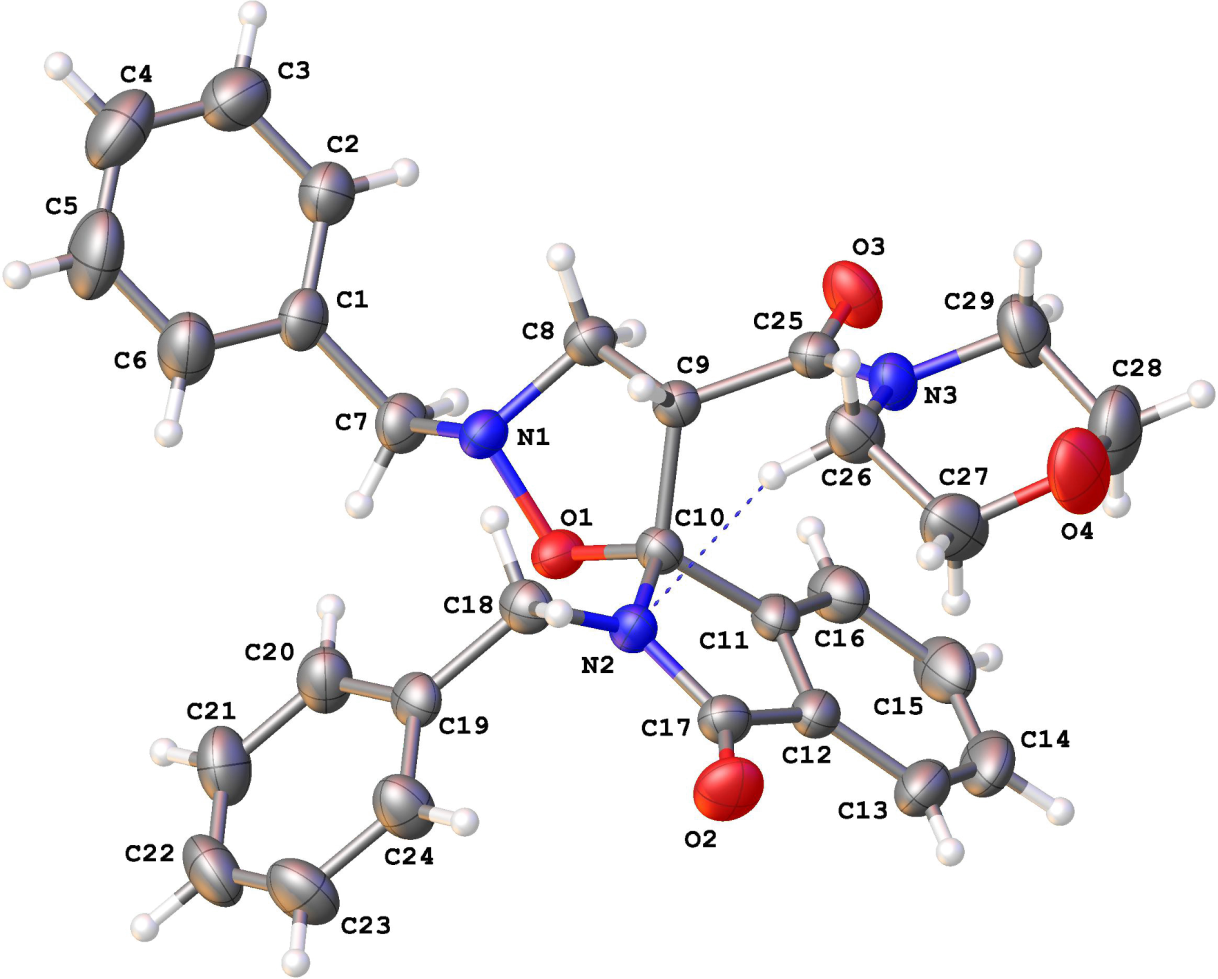
ORTEP drawing of the X-ray crystal structure of **7a** showing the model atomic numbering scheme (C10 and C9 correspond to the chiral centres C1 and C4’, respectively). Crystal packing is a racemate due to the centrosymmetric symmetry and in the picture the choice of the enantiomer is arbitrary. C: light blue, H: white, O: red, N: magenta. Probability of the ORTEP ellipsoids is set to 50%, whereas H size is arbitrary.

Furthermore, X-ray diffraction measurements confirm the structural assignment. Unfortunately, it was possible only to obtain a single crystal for the minor isomer **7** and the relative configuration, as *RS*/*SR*, at C10 and C9, respectively, is reported in Figure 4 [36].

In order to further evaluate the regio- and the stereochemical outcome of the cycloaddition reaction and to extend the potentiality of the synthetic process, dipolarophile **2a** was reacted with nitrones **5** under the same experimental conditions. However, only the starting isoindolinone was recovered. Also the use of more drastic reaction conditions, such as the use of *o*-xylene as a solvent and higher temperatures up to 140 °C failed, leading to the degradation of the nitrone and the isolation of the unaltered dipolarophile, so clearly indicating that the steric factors play a crucial role in the cycloaddition process (see computational data).

### Theoretical calculations

**Scheme 3:**
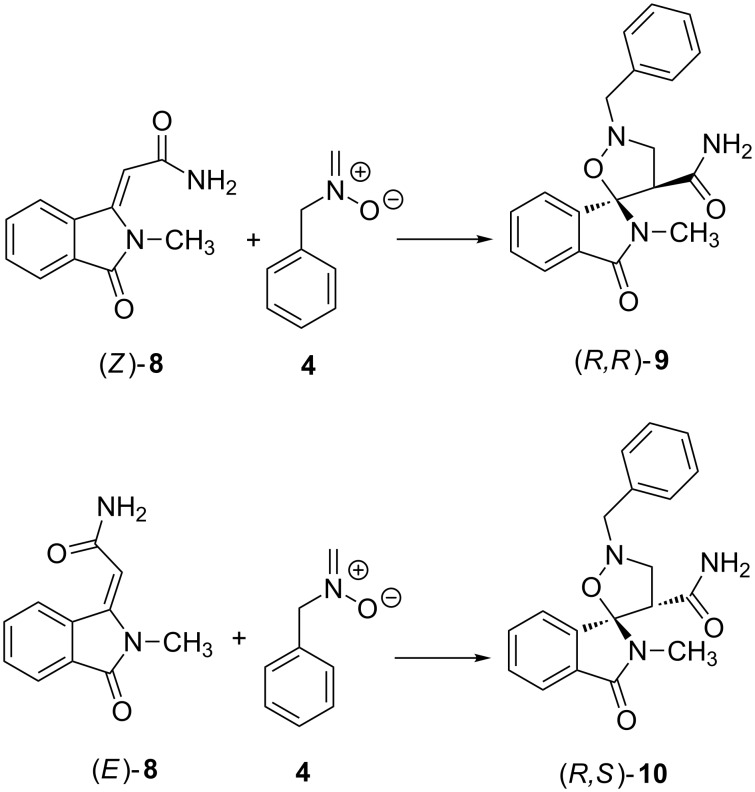
Reaction pathway.

The obtained results and stereochemical outcome of the 1,3-dipolar cycloaddition process have been rationalized through a mechanistic study on the basis of our expertise in the study of cycloaddition reactions and heterocyclic compounds [37–42]. Calculations were performed using the Gaussian09 program package, [43] through optimizations with the Thrular’s functional M06 [44] and 6-31+G(d,p) basis set. A simplified model, able to correctly mimic the system, was considered and the reaction between dipolarophile **8** (*Z* and *E*) and nitrone **4** (Scheme 3) was studied.

**Figure 5:**
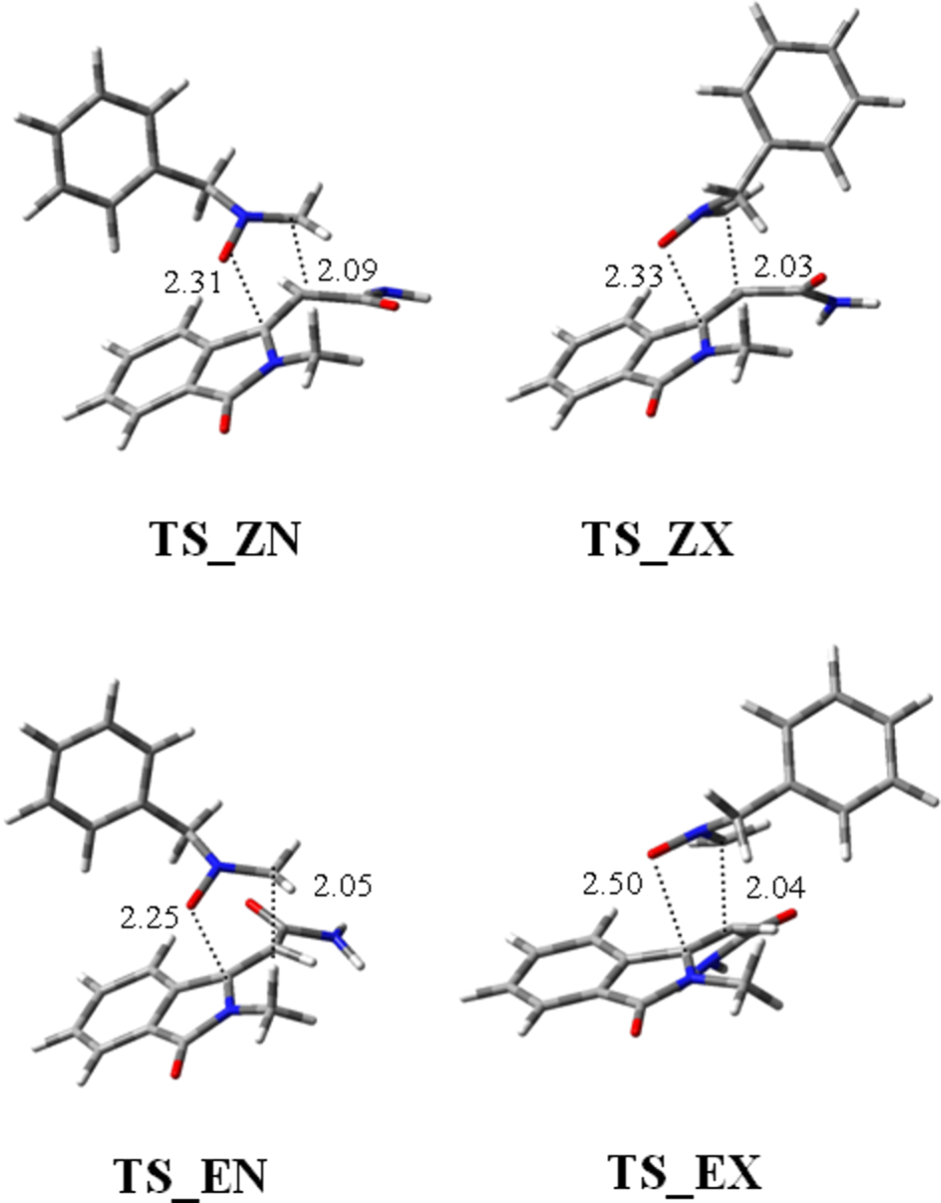
Three-dimensional plots of TSs of reaction of dipolarophiles (*Z*)-**8** and (*E*)-**8** with nitrone **4**. The labels *endo* N and *exo* E refer to the location of the nitrone *N*-benzyl in the formed five-membered ring on the same or opposite side of the phenyl ring of dipolarophiles. Distances between atoms involved in the forming bonds (Å) are reported in the 3D plots.

The reaction pathway was considered and the transition states leading to the (*R*,*R*)*-***9** or (*R*,*S*)-**10** adducts were modeled. Figure 5 shows the TSs (named *endo*, **N** or *exo*, **X**) for reaction of the two isomeric dipolarophiles (*Z*)-**8** and (E)-**8** with nitrone **4**, respectively. All the possible degrees of conformational freedom were considered, in particular the different orientations of the benzylic moiety. The possibility, in the *endo* TSs, of stacking interactions between aromatic rings, as hypothesized in the literature [45], was taken into account, but the corresponding geometries are too high in energy and evolve to the TSs reported in Figure 5.

**Figure 6:**
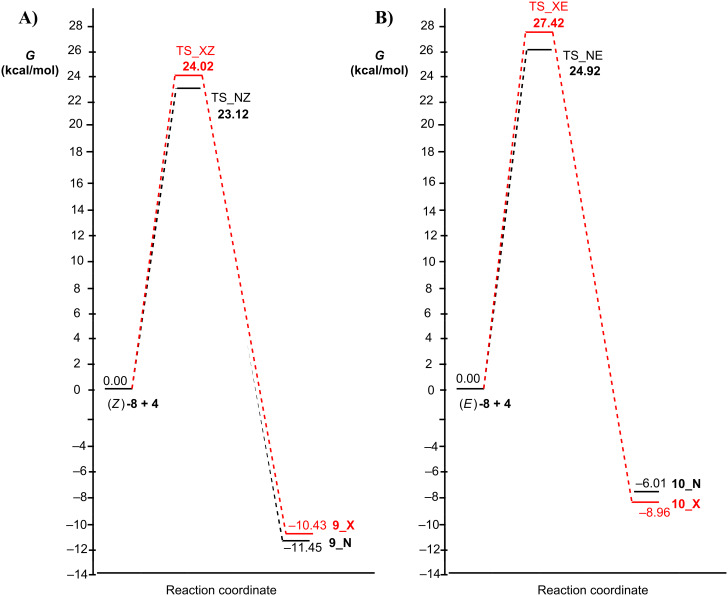
Free energy profiles for the cycloaddition reaction of the two isomers *Z* (A) or *E* (B) of dipolarophile **8** with nitrone **4**, considering both the *endo* (N, black) or *exo* (X, red) path.

The profiles of the four reactions are shown in Figure 6 and the percentages of the adducts derived from the TSs at 408 K are given in Table 2. The cycloadducts **9** and **10** are formed with a ratio of 92:8.

**Table 2:** Relative free energies of TSs and percentages of the corresponding adducts at 408 K of the reaction of dipolarophiles (*Z*)-**8** and (*E*)-**8** with nitrone **4**.

TS	Δ*G* (kcal/mol)	% (408 K)

ZX	24.02	22.8 (*R*,*R*)-**9**
ZN	23.12	69.3 (*R*,*R*)-**9**
EN	24.92	7.5 (*R*,*S*)-**10**
EX	27.42	0.3 (*R*,*S*)-**10**

Calculations supported the experimental data, showing that the adduct *RR* in the racemic mixture is the mainly obtained product. It is worthy pointing out that increasing the steric hindrance on the carbon atom of nitrone **5**, such as replacing one hydrogen atom with a methyl group, the energy barriers become significantly higher (about 30 kcal/mol), so the reaction is expected to be difficult.

### Biological tests

#### Cellular viability and proliferation

The synthesized compounds were assayed for their biological activity on three human cancer cell lines (the neuroblastoma SH-SY5Y, the HT-29 colorectal adenocarcinoma and the HepG2 hepatocellular carcinoma cells) treated for 24–72 h with the tested compounds. The MTS assay [46–47] showed a significant reduction in cellular viability in all cancer cell lines treated with compounds **6a–f** at concentrations ranging from 1 to 100 µM, when compared with respective controls. No significant effect in cellular viability in all cancer cell lines was found when the cells were exposed to the synthesized compounds for 24 and 48 h (data not shown). In particular, compound **6e** showed to be the most active derivative and displayed the greatest activity in the range of 9.41 to 21.58 µM. Furthermore, SH-SY5Y cell lines were more susceptible to treatment with **6e,** than the HT-29 and HepG2 cells. Thus, the other experiments have been performed using **6e** as model compound.

**Figure 7:**
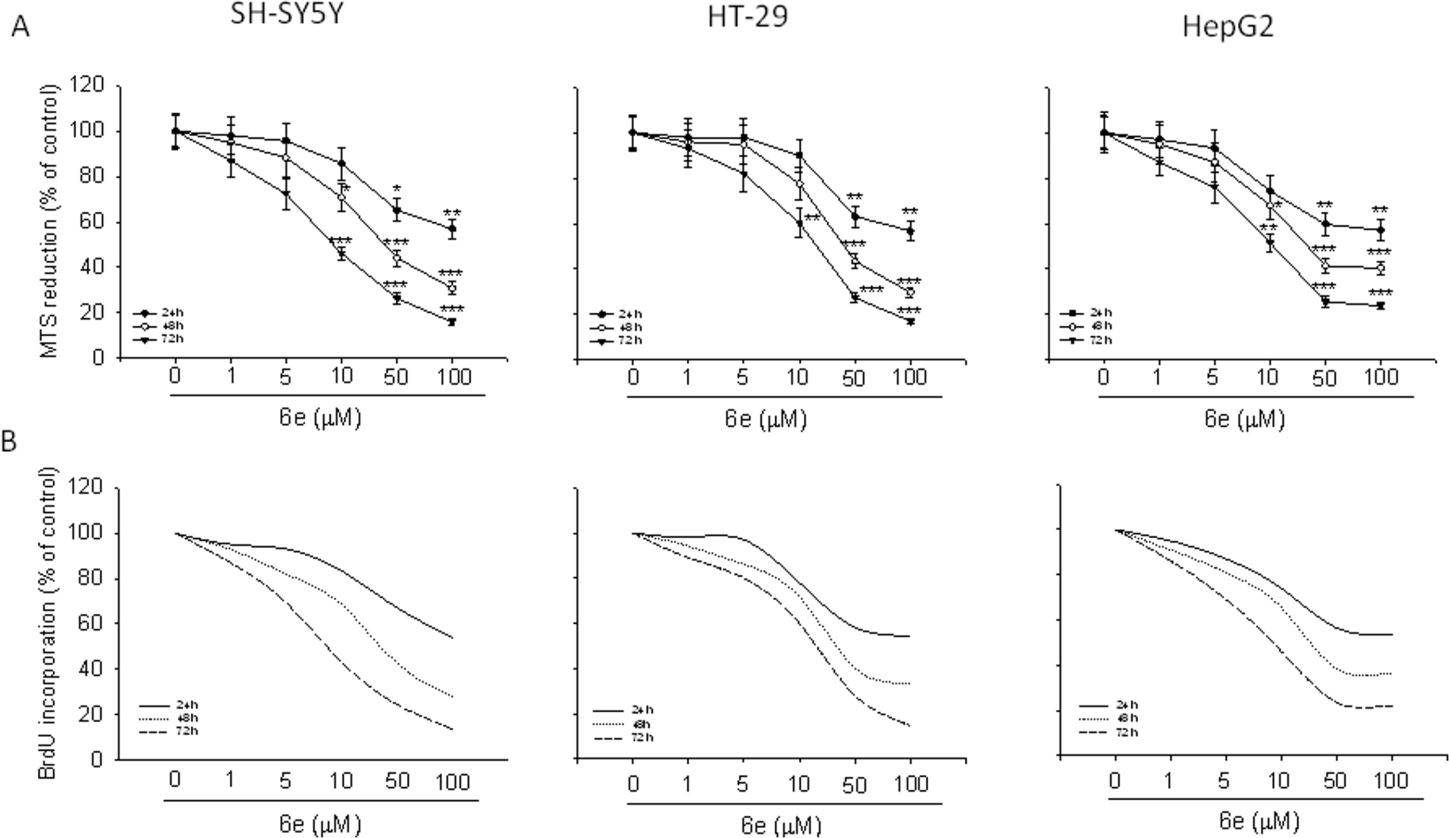
Compound **6e** reduces cancer cell proliferation. Treatment of SH-SY5Y, HT-29 and HepG2 cells with **6e** in a range from 1 to 100 µM for 24, 48 and 72 h reduced the growth rate in a time and concentration-dependent manner. The experiments were performed by the MTS assay (a) and BrdU test (b). Results are expressed as percentages of growth rates of treated cells compared to untreated cultures, and are the means ± SEM from of independent experiments performed in eightplicate (MTS assay) or in triplicate (BrdU test). *P < 0.05, **P < 0.01 and ***P < 0.001 vs untreated cells.

In general, all the synthesized compounds showed a certain degree of antiproliferative effect against all the examined cancer cells with a similar trend (see Supporting Information File 1, Figure S1). Noteworthy, compound **6e** exhibited superior activity with respect to other derivatives. As shown in Figure 7a, treatment of SH-SY5Y, HT-29 and HepG2 cells with **6e** ranging from 1 µM to 100 µM, for 24–72 h, reduced cell growth in all cancer cell lines. In particular, the maximal growth inhibitory effect of **6e** was reached after 72 h of incubation with the 100 µM concentration, corresponding to 72% in HepG2 (IC_50_ 10.50 µM), 83% and 84% in HT-29 (IC_50_ 21.58 µM) and SH-SY5Y (IC_50_ 9.41 µM) cell lines, respectively (*P* < 0.001 vs control). Significant reduction of cell proliferation was also observed when the cultures were exposed to **6e** for 24 hours (*P* < 0.01 vs control) and 48 (*P* < 0.001 vs control). Lesser, but still significant, an antiproliferative effect was also found treating the cells with **6e** at concentrations of 50, 10 and 5 µM for all time of exposure, while a concentration of 1 µM did not exert a significant antiproliferative effect.

Assessment of cell proliferation was also performed cytofluorimetrically by the BrdU assay, [48] obtaining results that reflect data from MTS test (Figure 7b).

#### Cytotoxic effect

**Figure 8:**
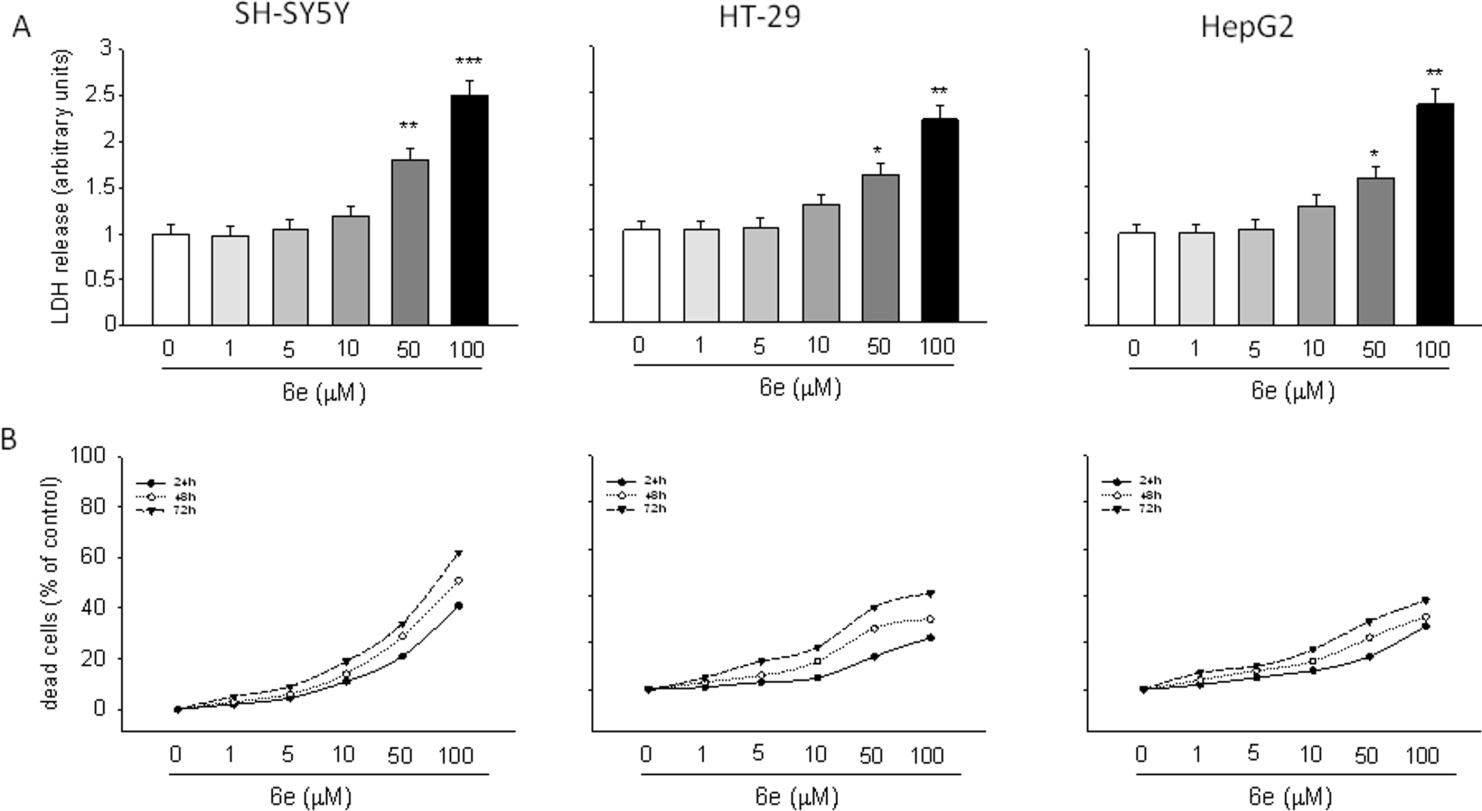
Cytotoxic effect of **6e**. The cytotoxic activity of **6e** was assessed in terms of both LDH release (a) and cell death (b). LDH levels are extrapolated as the values detected in untreated cells, which are arbitrarily expressed as 1. Cell death was reported as the percentage of dead cells vs untreated cultures point to 0. Data, expressed as mean ± S.E.M., represent the values obtained in three different sets of experiments made in triplicate. *P < 0.05, **P < 0.01 and ***P < 0.001 vs untreated cells.

The cytotoxic effect induced by **6a–f** was evaluated by an LDH assay [49], revealing that significant cytotoxicity was exerted only at the higher concentrations (50 and 100 µM; see Supporting Information File 1). Figure 8a shows that **6e** caused a significant increase of LDH release at 10, 50 and 100 µM concentration in all cell lines used in this study (*P* < 0.01 and *P* < 0.001 for SH-SY5Y cells and *P* < 0.05 and *P* < 0.01 for HT-29 and HepG2 cells). The LDH release was accompanied by a significant increase in cell death, as detected by flow cytometry through a propidium iodide assay (Figure 8b) [50–51].

#### Involvement of p53 in the pharmacological activity

Tumor suppressor p53 plays an important role in conserving genome stability by preventing its mutation. Normally, p53 is found at low levels because of its continuous proteasomal degradation promoted by MDM2. MDM2 inhibits p53 activity in two ways: i) by targeting p53 into the region of interaction with CBP/p300, thus preventing its transcriptional activity, and ii) by exporting p53 from the nucleus to the cytosol, acting as E3 ubiquitin ligase that marks p53 for degradation by the proteasome. Following DNA damage, p53 rises and becomes phosphorylated, thus translocates to nucleus where it binds DNA and activates expression of several proteins that arrest cell proliferation until the damage is repaired. If the damage is too severe, p53 trigs apoptosis which allows the elimination of damaged cells, also by its translocation in the mitochondria where it inhibits the activity of anti-apoptotic proteins. Many tumors overproduce MDM2 to impair p53 function, thus promoting cancerogenesis.

**Figure 9:**
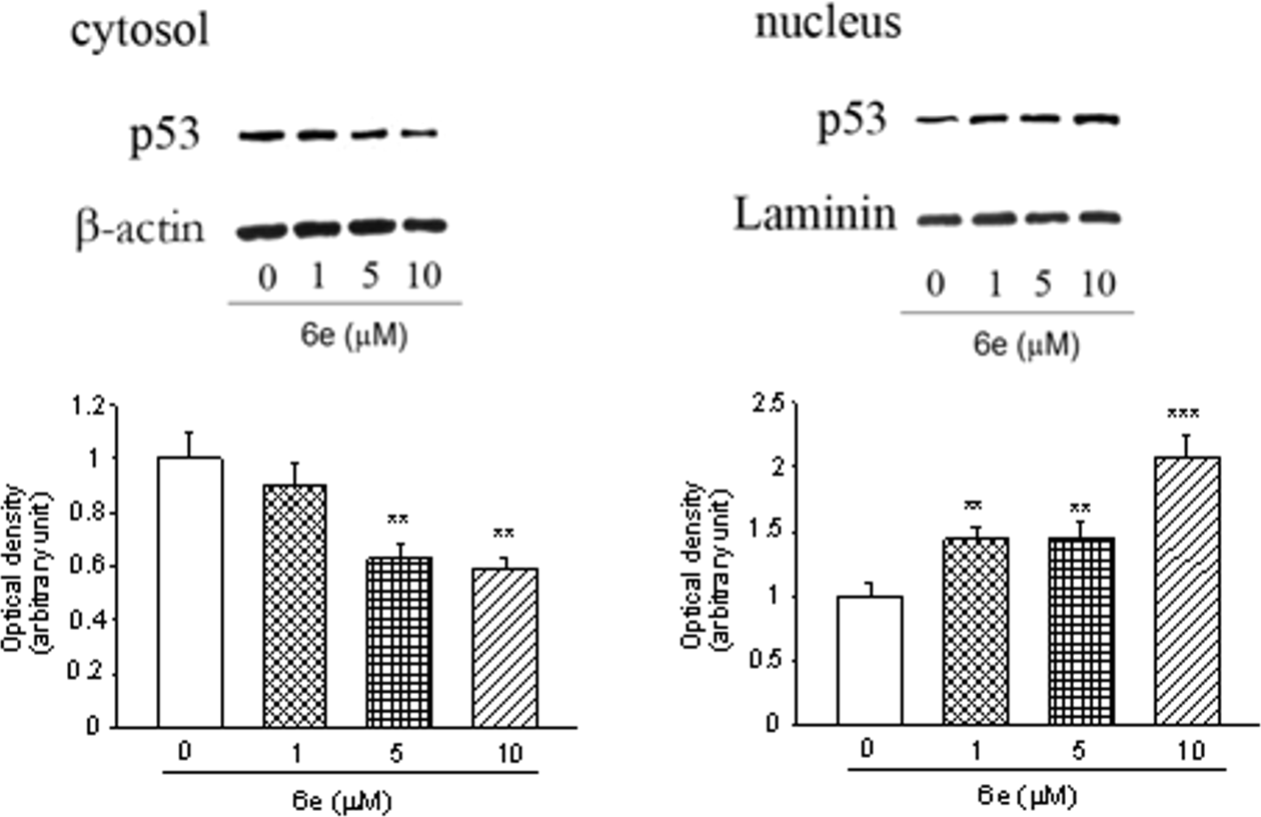
**6e** modulate the levels of p53 in SH-SY5Y cells. (a) The SH-SY5Y cells were treated for 24 h with the indicated concentration of **6e**, and then, both cytosolic and nuclear proteins were analyzed by Western blot for p53 protein. A representative immunoblot of three independent experiments is shown. (b) A densitometric analysis of autoradiographic bands collected from three separate experiments is shown. Levels of nuclear or cytosolic protein were normalized for laminin or β-actin, respectively. **P < 0.01 and ***P < 0.001 vs untreated cells.

In order to evaluate the possible involvement of p53 in the antiproliferative and cytotoxic effect of **6e**, we assessed the levels of p53, MDM2 and p21 by Western blot analysis [52]. We have chosen the SH-SY5Y cells because of their greatest sensitivity to this molecule in comparison to the other cultures employed in this study. The cells were treated for 72 h with **6e** at concentrations that do not induce any cytotoxic effects (1–10 µM). As shown in Figure 9, incubation with 5 and 10 µM concentration decreased the levels of p53 in the cytosol (*P* < 0.01 vs untreated cells) and increased those in the nucleus (*P* < 0.01 and *P* < 0.001 vs untreated cells), demonstrating its involvement in the anti-cancer effect elicited by **6e**.

**Figure 10:**
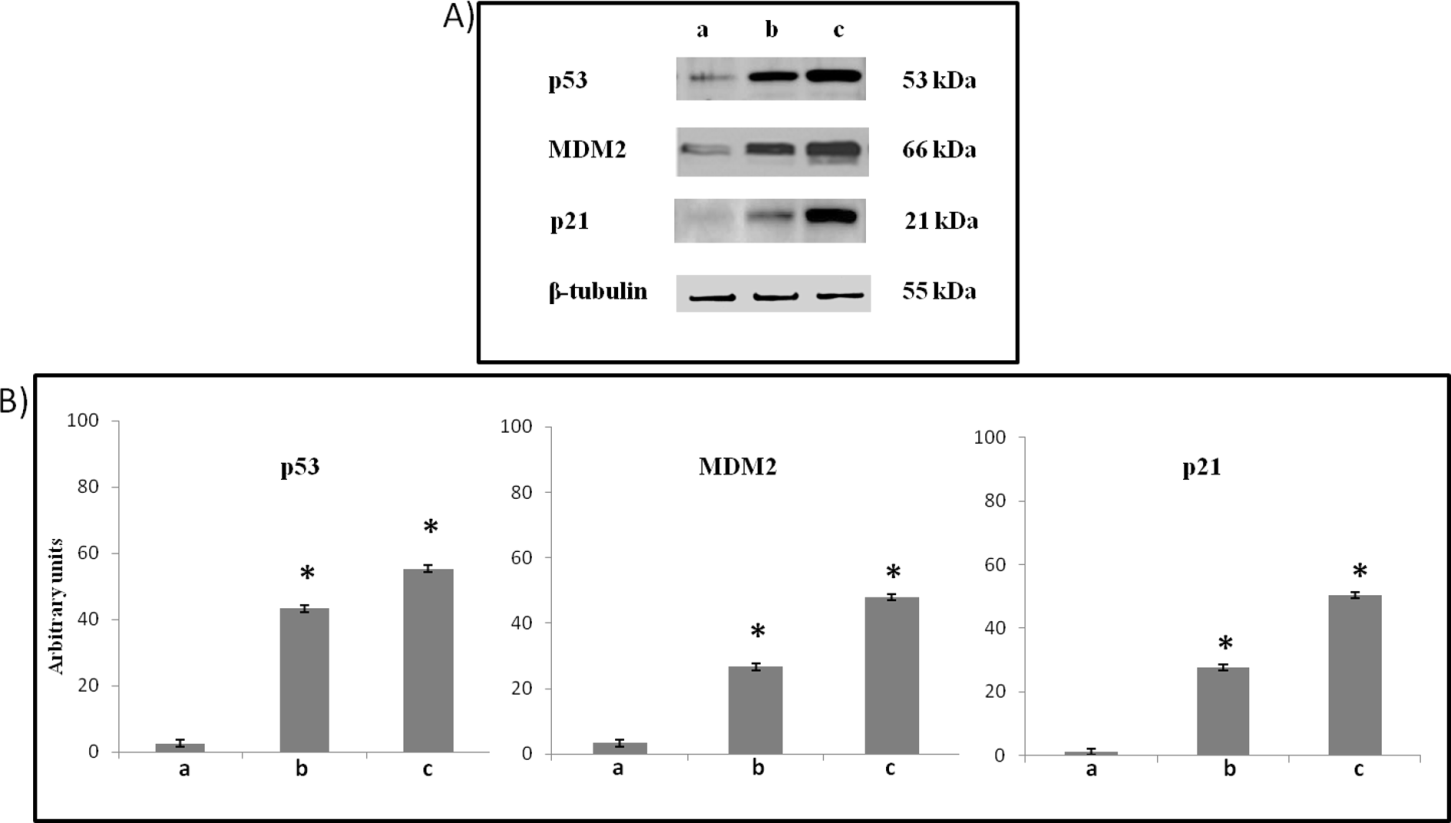
(A) Representative Western Blots and (B) semiquantitative analyses of p53, MDM2, p21 expression levels in total cellular lysates of SH-SY5Y cells non-exposed (a), exposed at 72 h to 10 μM **6e** (b) or 10 μM Nutlin-3 (c), performed after normalization with β-tubulin. Blots shown are representative ones of a Western Blot analysis of four experiments in duplicate. Results are expressed as the mean ± S.D. of the values of four experiments in duplicate. *P < 0.05, significant differences vs controls.

Treatment of the cells with **6e** leads to increased p53 protein expression, a compensatory increase in MDM2 expression, and activates p53-mediated apoptosis with an increase in p21 expression, when compared with the control. The effects appeared lower than it can be found in cells treared with Nutlin-3, a known MDM2-p53 antagonist [12] (Figure 10).

#### Effect of **6e** on apoptotic pathway activation

To elucidate whether **6e** might be related to apoptotic pathway, we studied by Western Blot analysis, caspase-3 and PARP cleavage, in SH-SY5Y cell line cultures.

**Figure 11:**
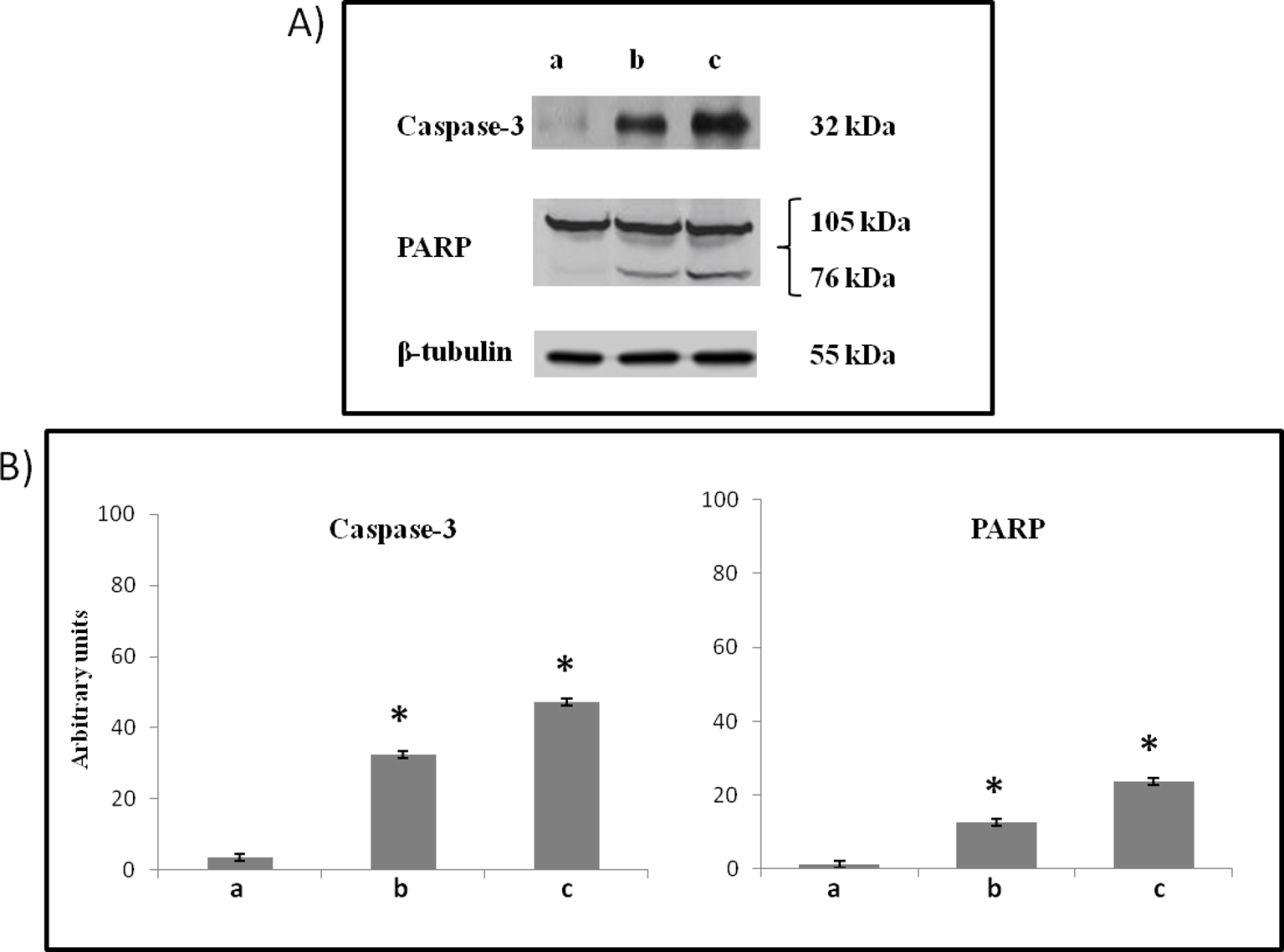
(A) Representative Western Blots and (B) densitometric analysis of caspase-3 and PARP cleavage in total cellular lysates of SH-SY5Y cells non-exposed (a), exposed at 72 h to 10 μM **6e** (b) or 10 μM Nutlin-3 (c), performed after normalization with β-tubulin. Blots shown are representative of Western blot analysis of four experiments in duplicate. Results are expressed as the mean ± SD of the values of four experiments in duplicate. *P < 0.05, significant differences vs controls.

A significant activation of caspase-3 and PARP cleavage in 10 µM SH-SY5Y-treated cells was found (Figure 11b), when compared with the untreated ones (Figure 11a), even if its effect is lower than found in Nutlin-3-treated cells (Figure 11c).

These set of experiments demonstrate that the exposure of SH-SY5Y cancer cell lines to 10 µM **6e** for 72 h was able to activate the apoptotic pathway.

#### Docking studies

To support the suggested interaction of synthesized compounds with MDM2, docking studies were applied, starting from the X-ray coordinates of the complex of the MI63-analogue with MDM2 [53–54]. The protein structure PDB ID 3LBL was chosen as the reference receptor because its ligand had high binding affinity and high resolution (1.6 Å). Docking studies were performed using AutoDock4.2 and both enantiomers of compounds **6a–f** were docked into the MDM2 binding site.

The docking protocol starts with the redocking of the MI63 analogue in the binding site to determine the lowest RMSD relative to the crystallographic pose. The ligand was successfully redocked with a RMSD of 0.59 Å. Determination of the single binding mode of spiro-isoxazolidin isoindolinone scaffold in the receptor/ligand complex was difficult because of the open and lipophilic nature of the p53 binding site on MDM2. Therefore, prediction of the possible binding mode was based on two energy types, i.e., the lowest binding energy of the largest cluster and the intermolecular energy (Table 3).

**Table 3:** Estimated lowest binding energy based on the largest number in cluster (Δ*G*) and intermolecular energy (*IE*).

Compound	Δ*G* (kcal/mol)	*IE* (kcal/mol)

MI63-analogue (redocked RMSD 0.59 Å)	−9.78	−**11.27**
**6a**	−8.91	−10.40
**6b**	−8.03	−9.82
**6c**	−7.95	−9.74
**6d**	−8.43	−10.20
**6e**	−8.39	−**11.92**
**6f**	−8.76	−9.95

Through the docking results analysis, we found out that spiro[isoindolin-isoxazolidin] derivatives efficiently bind to the surface of MDM2 only by hydrophobic interaction. In particular, the best docking results were obtained for the (*S*,*S*)-enantiomers and, in agreement with the biological evaluation, the compound (*S*,*S*)-**6e** has shown an intermolecular energy value comparable with that of the co-crystallized ligand (MI63 analogue).

In addition to a lowest binding energy and intermolecular energy of docking success, we have also performed a visual inspection to confirm that all the chosen docking poses reproduced the p53 residue (Phe19, Trp23 and Leu26) and the ligand moieties occupied the three main hydrophobic pockets of MDM2.

**Figure 12:**
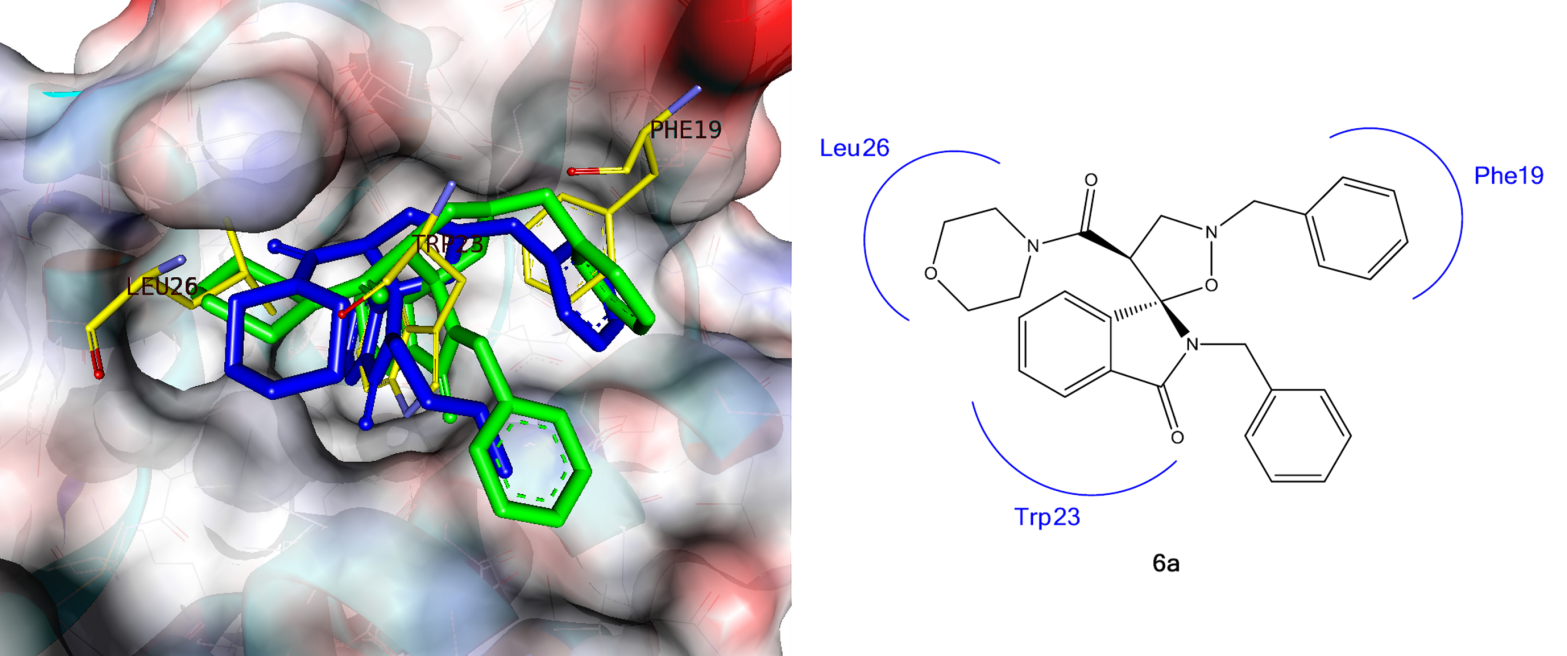
Compounds **6a** (green) and **6c** (blue) docked into MDM2 structure (PDB-ID: 3LBL) superimposed on the key amino acid side chain residues of the p53/MDM2 (yellow sticks, PDB-ID: 1YCR).

As shown in Figure 12, compounds **6a** and **6c** bind to MDM2 with a similar pose. Specifically, the isoindolinone moiety occupies the Trp23 pocket, the isoxazolidine ring projects its benzyl and morpholinamide groups into the Phe19 and Leu26 pockets respectively. Likewise compounds **6b**, **6d** and **6g**, did show a similar binding mode.

**Figure 13:**
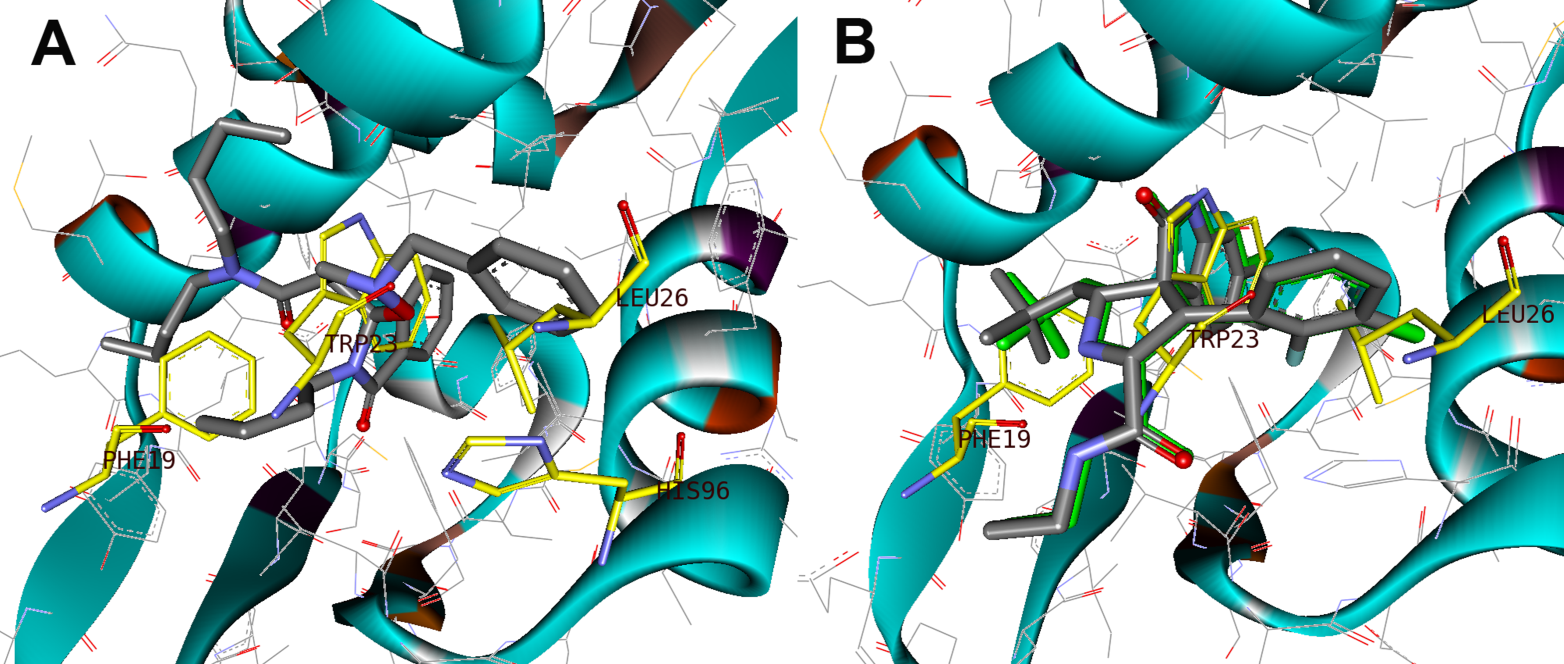
A) Compounds **6e** (green) docked into MDM2 structure (PDB-ID: 3LBL); His96 and p53 residue (yellow sticks, PDB-ID 1YCR) are included for reference. B) Co-crystal structure of MI63-analogue in MDM2 and redocked MI63-analogue (green sticks) superimposed on the key amino acid side chain residue of the p53/MDM2 (yellow sticks, PDB-ID: 1YCR).

Conversely, for spiro[isoxazolidin-isoindolinone] **6e**, the presence of the acyclic amide group on the isoxazolidine ring produces a 180° rotation of the isoindolinone core such that the benzyl group and dialkylamide occupy the Leu26 and Phe19 pockets, respectively. The reoriented binding mode, similar to the MI63 analogue, takes advantage of the π–π stacking interaction with His96 of MDM2 and the benzyl moiety of compound **6e**, and could be related to the greater stability of the complex ligand/MDM2 (Figure 13) and to the better biological activity.

## Conclusion

Spiro[isoindole-1,5-isoxazolidin]-3(2*H*)-ones **6a–f**, synthesized by 1,3-dipolar cycloaddition of *N*-benzylnitrone with isoindolin-3-methylene-1-ones, have shown interesting cytotoxic and antiproliferative activity on three human cancer cell lines, the neuroblastoma SH-SY5Y, the HT-29 colorectal adenocarcinoma and the HepG2 hepatocellular carcinoma cells. In particular, the most active compound **6e** shows an IC_50_ in the range of 9–22 μM: biological tests suggest that the antitumor activity could be linked to the inhibition of the protein–protein p53-MDM2 interaction. Docking measurements support the biological data. Further studies are needed to better clarify the role played by the new synthesized compounds in SH-SY5Y cancer cell lines as inhibitor of MDM2-p53 interaction.

## Experimental

**General:** Solvents and reagents are commercial. Microwave assisted synthesis was performed in a CEM Discover microwave oven using sealed reaction vessels. The temperature was monitored using a vertically focused IR temperature sensor. In order to have a homogenous system all the batches were started with a ramp time of 120 seconds and when the temperature program was completed a cooling period of 10 minutes was included. ESI-HRMS were determined with a Thermo Fischer Scientific LTQ Orbitrap XL. NMR spectra (^1^H NMR at 500 MHz, ^13^C NMR at 126 MHz) were recorded with Varian instruments and are reported in ppm relative to CDCl_3_ (7.26 ppm). Merck silica gel 60-F254 precoated aluminum plates have been used for thin-layer chromatographic separations. Flash chromatography was performed on Merck silica gel (200–400 mesh). Preparative separations were carried out by a MPLC Büchi C-601 by using Merck silica gel 0.040–0.063 mm. X-ray crystal structure determination of compound **6a** was performed at room temperature on a suitable single crystal, obtained by recrystallization from ether/dichloromethane 2:1, by a Bruker AXS K Apex II CCD diffractometer with Mo Kα radiation (λ = 0.71073 Å). The structure was solved with the SIR2011 [55] structure solution program using Direct Methods and were refined with the ShelXL [56] refinement package using Weighted Least Squares minimization. All compounds were determined to havea a purity >95% using a Shimadzu LC/MS/MS-8040 system (C18 column; eluting gradient 10–90% acetonitrile in water).

**Materials:** Nitrones **4**, **5** and dipolarophiles **2a–g** have been prepared according to known procedures [35,57–59].

**General 1,3-dipolar cycloaddition procedure.** A solution of **2a** (0.287 mmol) and nitrone **4** (0.287 mmol) in toluene (5 mL) was put in a sealed tube and irradiated under microwave conditions at 200 W, 110 °C, for 4 h (CEM Discover Microwave reactor). The removal of the solvent in vacuo afforded a crude material which, after flash chromatography purification by using as eluent a mixture of chloroform/cyclohexane/ethyl acetate 5:3:2, gave compounds **6a** and **7a**, as white amorphous mass in 60% yield. The ^1^H NMR spectrum shows the presence of (*R*,*R*)/(*S*,*S*)- and (*R*,*S*)/(*S*,R)-isomers respectively in 85:15 ratio. A single crystal of **7a** suitable for X-ray diffraction studies was obtained by slow diffusion of diethyl ether into a hot dichloromethane solution of isomeric mixture, followed by filtration.

**(1*RS*,4'*RS*)-2,2'-Dibenzyl-4'-(morpholine-4-carbonyl)spiro[isoindoline-1,5'-isoxazolidin]-3-one (6a):** White solid; mp 197–199 °C (Yield: 71 mg, 51%); IR (KBr) ν_max_: 1685, 1638 cm^−1^; ^1^H NMR (500 MHz, CDCl_3_) δ 7.82–7.78 (m, 1H), 7.62–7.47 (m, 5H), 7.43 (d, *J* = 7.1 Hz, 2H), 7.36 (t, *J* = 7.3 Hz, 2H), 7.33–7.27 (m, 4H), 5.21 (d, *J* = 14.7 Hz, 1H), 4.90 (d, *J* = 14.7 Hz, 1H), 4.25 (d, *J* = 13.0 Hz, 1H), 4.08 (d, *J* = 13.0 Hz, 1H), 3.87–3.79 (m, 1H), 3.76–3.67 (m, 1H), 3.53–3.44 (m, 1H), 3.30 (dt, *J* = 11.5, 4.5 Hz, 1H), 3.10–3.00 (m, 3H), 2.82–2.74 (m, 1H), 2.62–2.54 (m, 1H), 2.53–2.46 (m, 1H), 2.38–2.30 (m, 1H); ^13^C NMR (126 MHz, CDCl_3_) δ 167.8, 166.5, 141.19, 138.7, 136.4, 132.4, 131.2, 130.5, 129.5, 129.1, 128.8, 128.7, 128.0, 127.9, 124.7, 123.2, 97.3, 66.3, 65.8, 63.1, 60.0, 51.7, 44.9, 43.7, 42.2; HRMS–ESI (*m*/*z*): [M + H]^+^ calcd for C_29_H_30_N_3_O_4_, 484.2236; found, 484.2262.

**(1*RS*,4'*SR*)-2,2'-Dibenzyl-4'-(morpholine-4-carbonyl)spiro[isoindoline-1,5'-isoxazolidin]-3-one (7a):** White crystals, mp 185–187 °C (Yield: 12 mg, 9%); IR (KBr) ν_max_: 1680, 1642 cm^−1^; ^1^H NMR (500 MHz, CDCl_3_) δ 7.80 (d, *J* = 6.7 Hz, 1H), 7.62–7.46 (m, 4H), 7.45–7.20 (m, 10H), 5.05 (d, *J* = 11.6 Hz, 1H), 4.83 (d, *J* = 11.6 Hz, 1H), 4.25 (d, *J* = 13.0 Hz, 1H), 4.12–3.98 (m, 2H), 3.86–3.80 (m, 1H), 3.42–3.37 (m, 1H), 3.33–3.27 (m, 2H), 3.08–3.04 (m, 1H), 2.80–2.72 (m, 1H), 2.74–2.64 (m, 1H), 2.61–2.52 (m, 1H), 2.54–2.46 (m, 1H), 2.39–2.31 (m, 1H); ^13^C NMR (126 MHz, CDCl_3_) δ 167.6, 162.4, 132.1, 130.3, 129.2, 129.0, 128.9, 128.5, 128.4, 128.2, 127.8, 127.6, 127.3, 126.8, 125.0, 124.4, 123.0, 100.0, 66.1, 65.5, 62.9, 59.7, 53.5, 44. 7, 43.4, 41.9; HRMS–ESI (*m*/*z*): [M + H]^+^ calcd for C_29_H_30_N_3_O_4_, 484.2236; found, 484.2271.

**(1*RS*,4'*RS*)-2'-Benzyl-2-butyl-4'-(pyrrolidine-1-carbonyl)spiro[isoindoline-1,5'-isoxazolidin]-3-one (6b):** Yellow sticky oil (Yield: 69 mg, 48 %); IR (neat) ν_max_: 1685, 1640 cm^−1^; ^1^H NMR (500 MHz, CDCl_3_) δ 7.72–7.68 (m, 1H), 7.62–7.53 (m, 1H), 7.53–7.49 (m, 1H), 7.47–7.42 (m, 1H), 7.41–7.34 (m, 2H), 7.34–7.27 (m, 3H), 4.20 (d, *J* = 12.7 Hz, 1H), 4.00 (d, *J* = 12.7 Hz, 1H), 3.92–3.83 (m, 1H), 3.75–3.66 (m, 1H), 3.64–3.54 (m, 1H), 3.25–3.17 (m, 1H), 3.16–3.08 (m, 1H), 2.73–2.61 (m, 2H), 1.75–1.60 (m, 4H), 1.59–1.51 (m, 2H), 1.40–1.24 (m, 4H), 0.90 (t, *J* = 7.4 Hz, 3H); ^13^C NMR (126 MHz, CDCl_3_) δ 167.3, 166.3, 136.4, 131.9, 130.1, 129.4, 129.2, 128.5, 127.8, 124.7, 122.6, 97.6, 63.1, 59.5, 53.9, 46.1, 46.0, 40.6, 31.4, 25.9, 23.8, 20.7, 13.9; HRMS–ESI (*m*/*z*): [M + H]^+^ calcd for C_26_H_32_N_3_O_3_, 434.2444; found, 434.2471.

**(1*RS*,4'*RS*)-2'-Benzyl-2-butyl-4'-(morpholine-4-carbonyl)spiro[isoindoline-1,5'-isoxazolidin]-3-one (6c):** White solid, mp 122–124 °C (Yield: 54 mg, 38%); IR (KBr) ν_max_: 1687, 1632 cm^−1^; ^1^H NMR (500 MHz, CDCl_3_) δ 7.77–7.74 (m, 1H), 7.57–7.48 (m, 3H), 7.38–7.27 (m, 5H), 4.19 (d, *J* = 12.7 Hz, 1H), 4.00 (d, *J* = 12.7 Hz, 1H), 3.84 (s, 1H), 3.78–3.70 (m, 1H), 3.64–3.55 (m, 1H), 3.47–3.39 (m, 2H), 3.36–3.28 (m, 1H), 3.10–3.02 (m, 2H), 2.83–2.73 (m, 2H), 2.71–2.65 (m, 1H), 1.76–1.66 (m, 2H), 1.39–1.29 (m, 2H), 1.24–1.17 (m, 2H), 0.92–0.86 (m, 3H); ^13^C NMR (126 MHz, CDCl_3_) δ 167.1, 166.5, 141.1, 136.1, 132.0, 130.3, 129.2, 129.1, 128.3, 127.7, 124.5, 122.8, 97.1, 66.2, 65.8 62.9, 59.9, 51.6, 45.2, 42.1, 40.3, 31.1, 29.7, 20.6, 13.7; HRMS–ESI (*m*/*z*): [M + H]^+^ calcd for C_26_H_32_N_3_O_4_, 450.2393; found, 450.2422.

**(1*RS*,4'*RS*)-2'-Benzyl-2-butyl-4'-(piperidine-1-carbonyl)spiro[isoindoline-1,5'-isoxazolidin]-3-one (6d):** White sticky oil (Yield: 45 mg, 35%); IR (neat) ν_max_: 1684, 1631 cm^−1^; ^1^H NMR (500 MHz, CDCl_3_) δ 7.73–7.69 (m, 1H), 7.54–7.43 (m, 3H), 7.37–7.34 (m, 2H), 7.33–7.26 (m, 3H), 4.18 (d, *J* = 12.7 Hz, 1H), 4.13–4.06 (m, 1H), 3.99 (d, *J* = 12.7 Hz, 1H), 3.90–3.81 (m, 1H), 3.78–3.70 (m, 1H), 3.61–3.55 (m, 1H), 3.51–3.43 (m, 1H), 3.18–3.10 (m, 1H), 3.08–2.96 (m, 2H), 2.76–2.67 (m, 1H), 1.78–1.69 (m, 2H), 1.39–1.23 (m, 8H), 0.89 (t, *J* = 7.4 Hz, 3H); ^13^C NMR (126 MHz, CDCl_3_) δ 167.4, 166.1, 141.4, 136.4, 131.9, 130.1, 129.4, 128.4, 127.8, 124.6, 122.6, 97.4, 63.1, 60.4, 51.6, 45.9, 42.9, 40.4, 31.2, 25.8, 25.2, 24.1, 20.7, 13.9; HRMS–ESI (*m*/*z*): [M + H]^+^ calcd for C_27_H_34_N_3_O_3_, 448.2600; found, 448.2629.

**(1*R*,4'*R*)-2'-Benzyl-*N*,*N*,2-tributyl-3-oxospiro[isoindoline-1,5'-isoxazolidine]-4'-carboxamide (6e):** Yellow oil (Yield: 52 mg, 38%); IR (neat) ν_max_: 1705, 1686, 1628 cm^−1^; ^1^H NMR (500 MHz, CDCl_3_) δ 7.71–7.67 (m, 1H), 7.62–7.26 (m, 8H), 4.17 (d, *J* = 12.7 Hz, 1H), 4.10–4.03 (m, 1H), 3.99 (d, *J* = 12.7 Hz, 1H), 3.88–3.79 (m, 1H), 3.74–3.66 (m, 1H), 3.62–3.54 (m, 1H), 3.54–3.40 (m, 2H), 3.39–3.34 (m, 1H), 3.19–3.14 (m, 1H), 3.03–2.96 (m, 1H), 2.48–2.30 (m, 2H), 1.68–1.54 (m, 4H), 1.37–1.20 (m, 6H), 0.95–0.85 (m, 6H), 0.67 (t, *J* = 7.2 Hz, 3H); ^13^C NMR (126 MHz, CDCl_3_) δ 167.3, 166.8, 136.3, 131.9, 130.1, 129.5, 129.3, 128.5, 127.8, 127.7, 125.2, 122.6, 97.6, 63.1, 60.5, 51.6, 47.9, 47.1, 46.4, 45.3, 43.7, 40.1, 31.3, 30.8, 29.6, 28.9, 20.7, 20.2, 19.9, 14.0; HRMS–ESI (*m*/*z*): [M + H]^+^ calcd for C_30_H_42_N_3_O_3_, 492.3226; found, 492.3255.

**(1*RS*,4'*RS*)-2'-Benzyl-4'-(morpholine-4-carbonyl)-2-phenylspiro[isoindoline-1,5'-isoxazolidin]-3-one (6f):** Yellow oil (Yield: 91 mg, 65%); IR (neat) ν_max_: 1683, 1636 cm^−1^; ^1^H NMR (500 MHz, CDCl_3_) δ 7.88–7.82 (m, 1H), 7.66–7.60 (m, 2H), 7.58–7.44 (m, 3H), 7.44–7.28 (m, 8H), 4.18 (d, *J* = 13.0 Hz, 1H), 4.10–3.96 (m, 1H), 3.78–3.72 (m, 1H), 3.62–3.57 (m, 1H), 3.56–3.49 (m, 1H), 3.49–3.44 (m, 1H), 3.44–3.39 (m, 1H), 3.39–3.32 (m, 2H), 3.24–3.05 (m, 3H), 2.86–2.79 (m, 2H); ^13^C NMR (126 MHz, CDCl_3_) δ 166.9, 166.5, 136.2, 134.7, 133.1, 132.9, 131.0, 130.5, 129.9, 129.4, 129.0, 128.8, 128.4, 128.0, 127.8, 125.0, 123.6, 101.5, 66.9, 66.5, 66.0, 45.4, 42.45; HRMS–ESI (*m*/*z*): [M + H]^+^ calcd for C_28_H_28_N_3_O_4_, 470.2080; found, 470.2052.

**(1*RS*,4'*SR*) 2'-Benzyl-2-*tert*-butyl-4'-(morpholine-4-carbonyl)spiro[isoindoline-1,5'-isoxazolidin]-3-one (7g):** Yellow oil (Yield: 14 mg, 10%); IR (neat) νmax: 1681, 1629 cm^−1^; ^1^H NMR (500 MHz, CDCl_3_) δ 7.68 (d, *J* = 7.4 Hz, 1H), 7.53–7.35 (m, 5H), 7.34–7.23 (m, 3H), 4.30–4.11 (m, 4H), 3.53–3.39 (m, 2H), 3.33–3.17 (m, 4H), 3.05–2.95 (m, 1H), 2.95–2.86 (m, 1H), 2.73–2.65 (m, 1H), 1.70 (s, 9H); ^13^C NMR (126 MHz, CDCl_3_) δ 169.76, 136.12, 132.16, 129.71, 129.10, 128.46, 127.65, 122.20, 100.19, 66.49, 65.91, 57.79, 53.10, 45.81, 42.45, 29.68; HRMS–ESI (*m*/*z*): [M + H]^+^ calcd for C_26_H_32_N_3_O_4_, 450,2393; found, 450.2371.

## Supporting Information

File 1 Biological tests, ^1^H and ^13^C NMR spectra of all new compounds, computational methods and X-ray data.
