# Supplementary material for: Synthesis of spiro[isoindole-1,5’-isoxazolidin]-3(2H)-ones as potential inhibitors of the MDM2-p53 interaction
Source: Beilstein J Org Chem. 2016 Dec 20;12:2793–807. doi: 10.3762/bjoc.12.278 (PMC5238597; doi:10.3762/bjoc.12.278)
Supplement: File 1 — Biological tests, 1H and 13C NMR spectra of all new compounds, computational methods and X-ray data. [file Beilstein_J_Org_Chem-12-2793-s001.pdf]

Supporting Information
for
Synthesis of spiro[isoindole-1,5'-isoxazolidin]-3(2*H*)-ones as potential inhibitors of the MDM2-p53 interaction

Salvatore V. Giofrè*[,§,1], Santa Cirmi[1], Raffaella Mancuso[2], Francesco Nicolò[3], Giuseppe Lanza[4], Laura Legnani[5], Agata Campisi[4], Maria A. Chiacchio[4,5], Michele Navarra[1], Bartolo Gabriele[2] and Roberto Romeo[*,¶,1]

Address: [1]Dipartimento di Scienze Chimiche, Biologiche, Farmaceutiche e Ambientali, Via S.S. Annunziata, 98168 Messina, Italy, [2]Dipartimento di Chimica e Tecnologie Chimiche, Università della Calabria, Via P. Bucci, 12/C, 87036 Arcavacata di Rende (CS), Italy, [3]Dipartimento di Scienze Chimiche, Biologiche, Farmaceutiche e Ambientali, Università di Messina, Viale F. Stagno d'Alcontres 31, 98166 Messina, Italy, [4]Dipartimento di Scienze del Farmaco, Università di Catania, Viale A. Doria, 95100 Catania, Italy and [5]Dipartimento di Chimica, Università di Pavia, Via Taramelli 12, 27100 Pavia, Italy

Email: Salvatore V. Giofrè - sgiofre@unime.it; Roberto Romeo - robromeo@unime.it

*Corresponding Author

[§]Phone: (+39) 090-6766566. Fax: (+39) 090-6766562.

[¶]Phone: (+39) 090-356230. Fax: (+39) 090-6766562

Biological tests, ^{1}H and ^{13}C NMR spectra of all new compounds, computational methods and X-ray data

Table of Contents

Biological tests S3

Figure S1 S7

^{1}H and ^{13}C NMR S9

M06/6-31+G(d,p) Free energies and cartesian coordinates S18

X-ray crystallographic data of **7a** S26

Biological tests

Cell culture and treatment with drugs

The pharmacological properties of the synthesized compounds were tested on three human cancer cell lines: the neuroblastoma SH-SY5Y, the HT-29 colorectal adenocarcinoma and HepG2 hepatocellular carcinoma cells. All cell lines were obtained originally from ATCC (Rockville, MD, USA) and were grown in monolayer at 37 °C with 5% CO_2 humidified atmosphere. The culture medium was a RPMI supplemented with 10% (v/v) heat-inactivated fetal bovine serum, L-glutamine (2 mM), sodium pyruvate (1 mM), penicillin (100 lU/mL) and streptomycin (100 mg/mL). All reagents were from Gibco (Life Technologies, Monza, Italy).

Compound were solubilized in dimethylsulfoxide (DMSO) at a 100 mM concentration (stock solutions) and then stored in aliquot at −20 °C. Drugs were diluted in culture media to the desired concentration just prior the use. The same DMSO concentrations, used to dissolve the compounds to the final concentration of 100 mM, served as vehicle control (0.1% DMSO in culture medium).In comparison with untreated cultures, DMSO 0.1% did not exert any significant influence on any parameters analyzed in this study (data not shown).

Colorimetric assays to evaluate the proliferation and cytotoxicity of cell culture

Cell growth was evaluated byCellTiter 96® AQueous One Solution Cell Proliferation Assay (Promega, Milan, Italy), a colorimetric method for determining the number of viable cells in proliferation assays. This method is based on the reduction of [3-(4,5-dimethylthiazol-2-yl)-5-(3-carboxymethoxyphenyl)-2-(4-sulfophenyl)-2*H*-tetrazolium (MTS) tetrazolium compound by viable cells to generate a colored formazan product that is soluble in cell culture media. This conversion is carried out by NAD(P)H-dependent dehydrogenase enzymes in metabolically active cells. Briefly, the cells were plated onto 96-well plates at a density of 5×10^3 cells/well (SH-SY5Y and HT-29 cells) or 6×10^3 cells/well (HepG2 cells). On the next day, the growth medium was replaced with fresh medium containing the spiro[isoindolinisoxazolidine] compounds at a concentration ranging from 1 to 100 µM. After 24, 48 and 72 h of incubation, the plates were centrifuged at 1200 rpm for 10 min. Next, the supernatant was removed and 100 µL of fresh medium without phenol red containing MTS was added to each well (final concentration of MTS was 0.33 mg/mL). Then, the plates were returned in the incubator for 3 h. The solubilized formazan product was spectrophotometrically quantified with a microplate spectrophotometer (iMark™ microplate

reader, Bio-Rad Laboratories, Milan, Italy) at a wavelength of 490 nm. Results are expressed as percentages of MTS reduction in untreated cultures (Romeo R. et al., 2014).

Cytotoxicity was assessed by using a commercial kit that measure lactate dehydrogenase (LDH) in the culture media (cytotoxicity assay kit, Cayman Chemical Company, Ann Arbor, Michigan, USA), as described (Giofrè S.V. et al., 2015). Lactate dehydrogenase is a cytosolic enzyme which is released into the culture medium when the plasma membrane is damaged, therefore it can be considered a marker of cytotoxicity. The released LDH can be quantified by a coupled enzymatic reaction. First, LDH catalyzes the conversion of lactate to pyruvate via reduction of NAD^+ to NADH, and second, diaphorase uses NADH to reduce a tetrazolium salt to a red formazan product. Therefore, the level of formazan formation is directly proportional to the amount of released LDH in the medium. SH-SY5Y, HT-29 and HepG2 cells were seeded at a density of 10×10^3 cells/well in 96-well plates and 24 h later the culture medium was substituted with fresh medium containing the tested compounds (1, 5, 10, 50 and 100 µM) for additional 24 h. Then, plates were centrifuged at 400 g for 5 min and 100 µl of supernatant from each well were transferred to corresponding wells on a new plate. Later, freshly prepared 100 µL LDH reaction solution was added to each well, and the plate incubated on an orbital shaker for 30 min at room temperature. The absorbance at 490 nm was quantified spectrophotometrically (iMark™ microplate reader). LDH levels are extrapolated as the values detected in untreated cells, which are arbitrarily expressed as 1.

Evaluation of cell growth and cytotoxicity by flow cytometer analyses

The 5-bromo-2'-deoxyuridine (BrdU) is a fluorescence-based test that can monitor cell division by evaluating DNA synthesis through thymidine incorporation. BrdU is a synthetic nucleoside analogue of thymidine, commonly used in the detection of proliferating cells. BrdU can be incorporated into the newly synthesized DNA of replicating cells, substituting for thymidine (Thy) during DNA replication. Antibodies specific for BrdU can be used to detect the incorporated Thy, thus indicating cells that were actively replicating their DNA (Visalli G. et al. 2014). The BrdU assay was performed using a commercial kit (BrdU Staining Kit, eBioscences, Inc., San Diego, CA, USA) according to the manufacturer’s protocol with some modifications. SH-SY5Y, HT-29 and HepG2 cells were seeded in 6-well plates at a density of 1×10^5 cells/well. After 24 h, the growth medium was replaced with fresh medium containing or not compound **6e** at a concentration ranging from 1 to 100 µM or DMSO (0.1%). After 24, 48 and 72 h of incubation, the plates were centrifuged at $350 \times g$ for 5 min, the supernatant was changed with fresh medium containing 10 µM of BrdU and

the cells were incubated for additional 24 h. Later, the cells were harvested, washed and stained with 1 mL/sample of DNase I/Staining Buffer for 1 h at 37 °C in the dark. Then, the cells were washed and incubated for 30 min at room temperature in the dark with 5 µL of Anti-BrdU fluorochrome conjugated antibody per sample. Finally, cells were washed and acquired on a Novocyte 2000 flow cytometer (ACEA Bioscences, Inc., San Diego, California, USA). Results are expressed as percentages of BrdU incorporation in untreated cultures.

Propidium iodide (PI) is a fluorescent dye that intercalates into double-stranded nucleic acid. It is excluded by viable cells, but can penetrate through damaged cell membranes of dead cells. Therefore, it is widely used to evaluate cell death. SH-SY5Y, HT-29 and HepG2 cells were seeded at density of 50 x 10^3cells/well in 24-well plates. After 24 h, the growth medium was replaced with fresh medium containing or not compound **6e** (1–100 µM) or DMSO (0.1%) and the cells were incubated for 24–72 h. Then, supernatants were collected, the cells were trypsinized and pooled with corresponding supernatants, washed and stained with PI (3 µg mL^{-1}; 15 min at 4 °C) in PBS. Dead cells, stained with the DNA intercalating probe, were cytofluorimetrically counted measuring the emission signals (Ferlazzo N. et al., 2015). Data are expressed as percentages of PI positive cells in untreated cultures.

Western blotting analysis for cytosolic and nuclear proteins.

SH-SY5Y cells were seeded at density of 5 × 10^5 in 6-well plates, treated for 24 h with different concentration of compound **6e** in a range of 1 to 100 µM and then processed following Ferlazzo and coworkers (2016). Briefly, the cells were washed with ice-cold PBS and lysed in a buffer containing: 0.32 M sucrose, 10 mM Tris-HCl, pH 7.4, 1 mM EGTA, 2 mM EDTA, 5 mM NaN_3, 10 mM 2-mercaptoethanol, 50 mM NaF and protease inhibitor (Roche Diagnostics Corporation, Indianapolis, USA). The homogenates were chilled on ice for 20 min, centrifuged at 9600 × *g* at 4 °C for 1 min and then the supernatant (cytosolic extract) was collected. Following, pellets were suspended in a lysis buffer containing 0.1% Triton X-100, 150 mM NaCl, 10 mM Tris-HCl, pH 7.4, 1 mM EGTA, 1 mM EDTA and protease inhibitors (Roche Diagnostics Corporation), kept on ice for 30 min and centrifuged at 9600 × *g* at 4 °C for 10 min. The supernatant (nuclear extract) was collected and stored at −80 °C until use. Protein concentrations were determined by using a Bio-Rad Protein Assay (Bio-Rad Laboratories) using BSA as standard. Proteins were separated on sodium dodecyl sulfate polyacrylamide gel electrophoresis (SDS-PAGE) and transferred onto PVDF transfer membrane (Immobilon-P PVDF, Merck Millipore, Darmstadt, Germany), blocked with PBS

containing 5% non-fat dried milk at room temperature for 1 h. Then, membranes were incubated at 4 °C overnight with the following antibodies: a mouse monoclonal anti-p53 antibodies (1:500; Abcam, Cambridge, UK), a rabbit polyclonal anti-laminin (1:1000; Abcam), a rabbit polyclonal anti-β-actin (1:1000; Cell Signaling Technology, Beverly, MA, USA). Later, membranes were incubated with horseradish peroxidise-conjugated goat anti-rabbit or anti-mouse IgG secondary antibodies (Abcam) at room temperature for 1 h. Protein bands were visualized using an enhanced chemiluminescence system (Luminata™ Forte, Western HRP substrate; Millipore), acquired with ChemiDoc™ MP System (Bio-Rad Laboratories) and quantified with the ImageJ software.

Western Blot analysis for expression levels of p53, p21, MDM2, caspase-3 and PARP

Untreated and treated SH-SY5Y with 10 µM of **6e** were harvested in cold PBS, collected by centrifugation, and resuspended in a homogenizing buffer with 50 mM Tris-HCl (pH 6.8), 150 mM NaCl, 1 mM EDTA, 0.1 mM phenylmethylsulfonyl fluoride (PMSF; Sigma), and 10 µg/mL of aprotinin, leupeptin, and pepstatin and sonicated on ice [Campisi A, Spatuzza M, Russo A, Raciti G, Vanella A, Stanzani S, Pellitteri R. *Neurosci Res* 2012, 72:89–295; Pellitteri R, Bonfanti R, Spatuzza M, Cambria MT, Ferrara M, Raciti G, Campisi A. *Mol Neurobiol.* 2016, 53: 1-10]. The protein concentration of the homogenates was then diluted to 1 mg/mL with reducing stop buffer (0.25 M Tris-HCl, 5 mM EGTA, 25 mM dithiothreitol, 2% SDS, and 10% glycerol with bromophenol blue as the tracking dye). Proteins were separated on 4–16% SDS–polyacrylamide gradient precast-gels and transferred to nitrocellulose membranes. Blots were blocked overnight at 48 °C with 5% non-fat dry milk dissolved in 20 mM Tris-HCl (pH 7.4), 150 mM NaCl, and 0.5% Tween 20. The expression levels of p53, p21, MDM2, caspase-3, PARP and β-tubulin were detected by incubation for 1 h with the respective monoclonal anti-mouse against each protein (1:1,000 in PBS), followed by incubation for 1 h with horseradish peroxidase–conjugated anti-mouse IgG (1:1,500 in PBS). The expression of each protein was visualized by a chemiluminescence (ECL) kit after autoradiography film exposure. Densitometric analysis was performed after normalization with anti-rabbit β-tubulin (1:1000, mAb 2128, Cell Signaling, EuroClone). Protein bands were visualized using an enhanced chemiluminescence system (Luminata™ Forte, Western HRP substrate; Millipore), acquired with ChemiDoc™ MP System (Bio-Rad Laboratories) and quantified with the ImageJ software.

Statistical analysis

Data were expressed as mean ± S.E.M. and statistically evaluated for differences using one-way analysis of variance (ANOVA), followed by Tukey–Kramer multiple comparisons test (GrafPAD Software for Science).

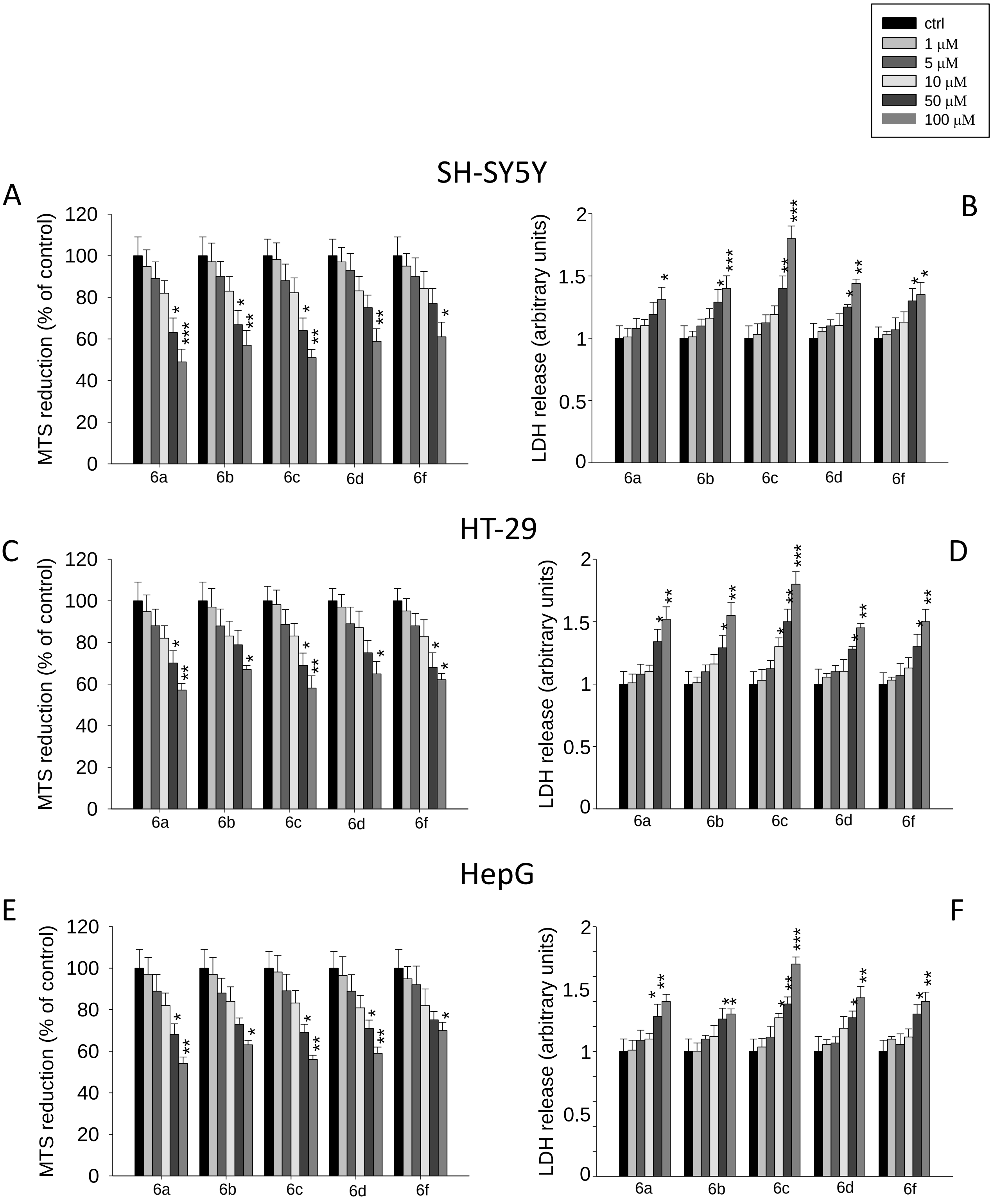

Figure S1. Effect of spiro[isoindolinisoxazolidine] compounds on cell growth and LDH release. The SH-SY5Y, HT-29 and HepG2 cells were exposed to increasing concentrations of **6a**, **6b**, **6c**, **6d** and **6f** as described in materials and methods. Herein are presented data of MTS cell proliferation assay performed after 72 h of incubation with the tested compounds at the indicated concentrations (A-C-E). Results are expressed as percentages of growth rates of treated cells compared to untreated cultures. The cytotoxic effect was evaluated in terms of LDH release after 24 h of exposure to synthetized molecules and are extrapolated as the values detected in untreated cells which are arbitrarily expressed as 1 (B-D-F). Results are the means ± SEM of three independent experiments performed in eightplicate (MTS assay) or in triplicate (LDH test). $^{*}P<0.05$, $^{**}P<0.01$ and $^{***}P<0.001$ *vs* untreated cells.

^{1}H NMR (500 MHz, $CDCl_3$) of compound **6a**

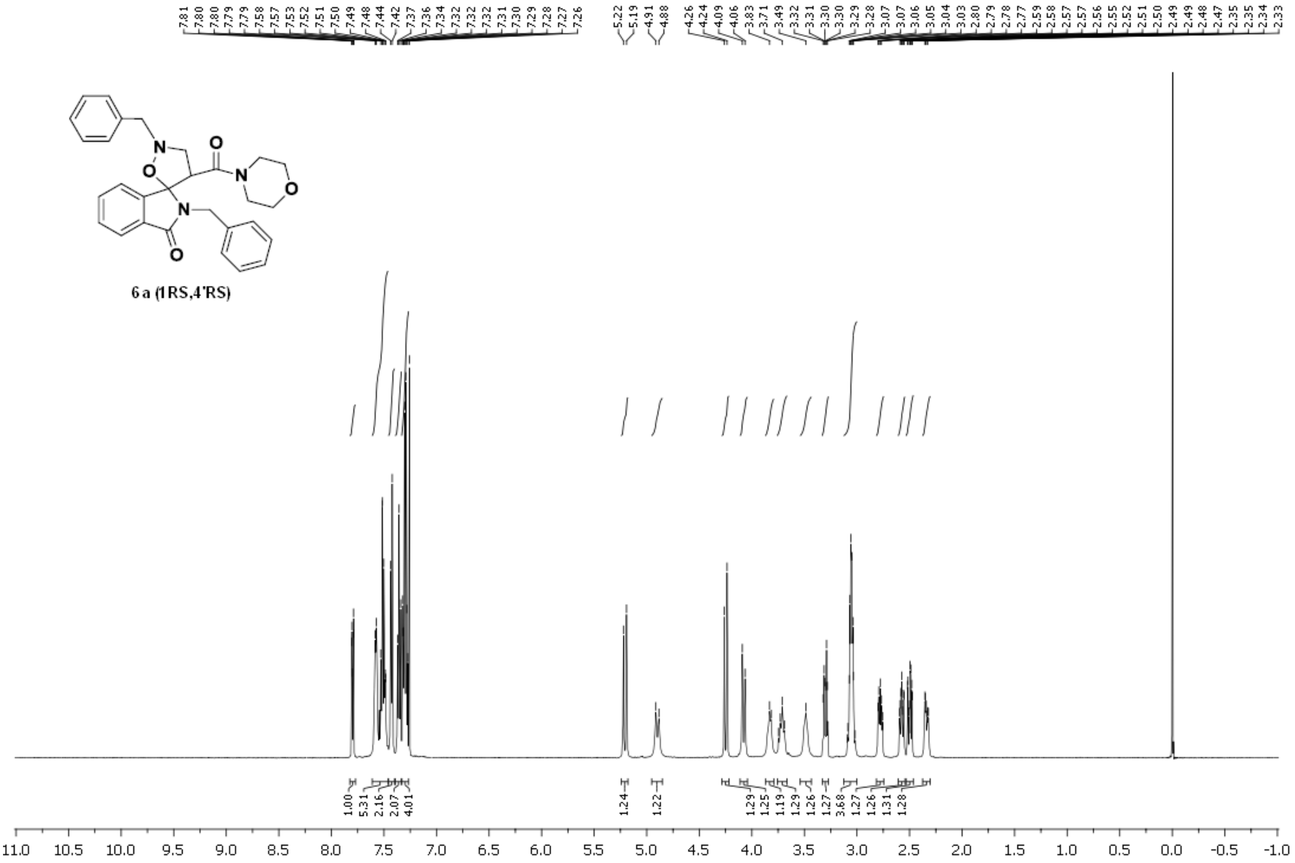

^{13}C NMR (125 MHz, $CDCl_3$) of compound **6a**

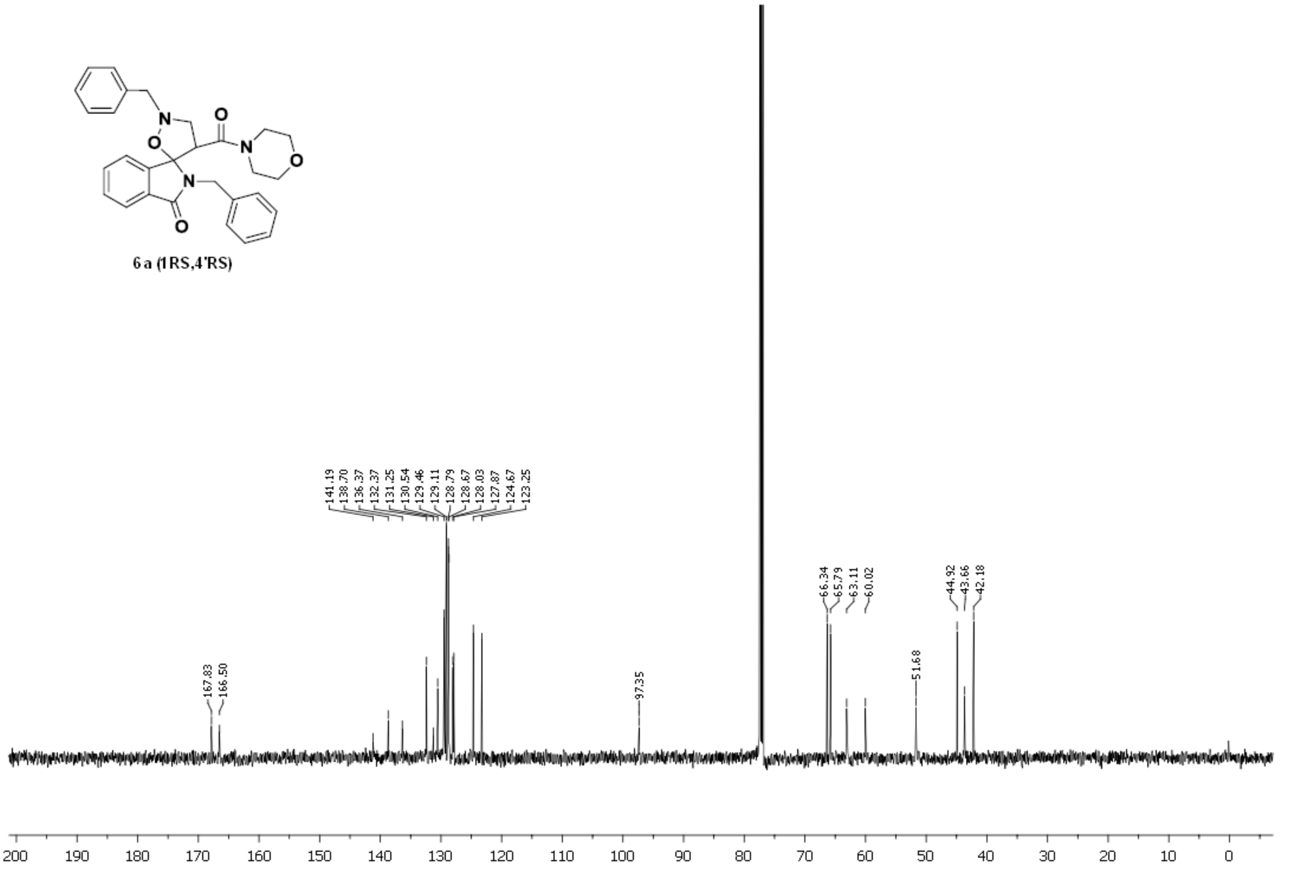

COSY and HSQC of compound **6a**

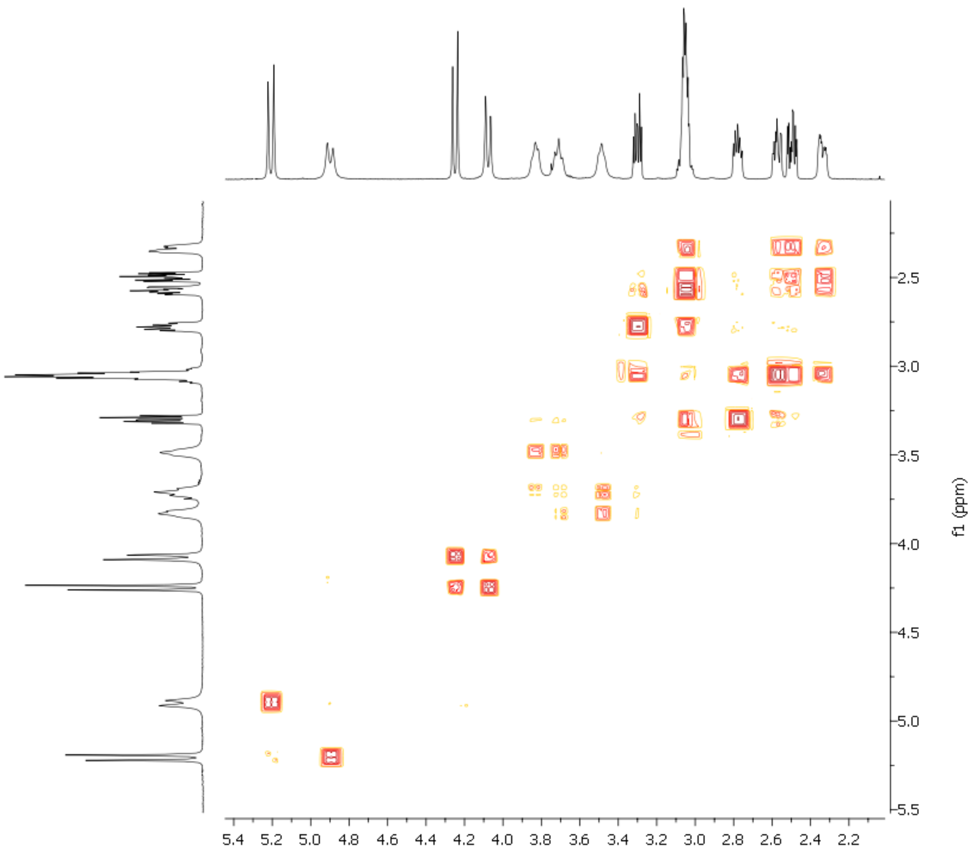

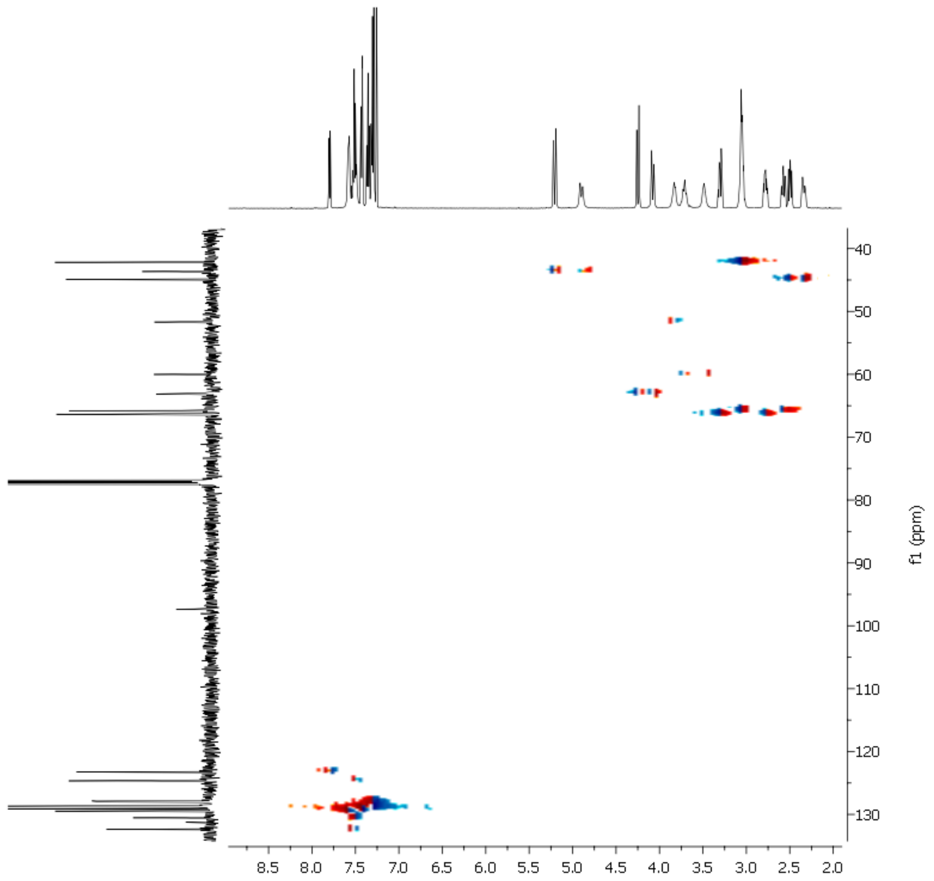

^{1}H NMR (500 MHz, $CDCl_3$) of compound **6b**

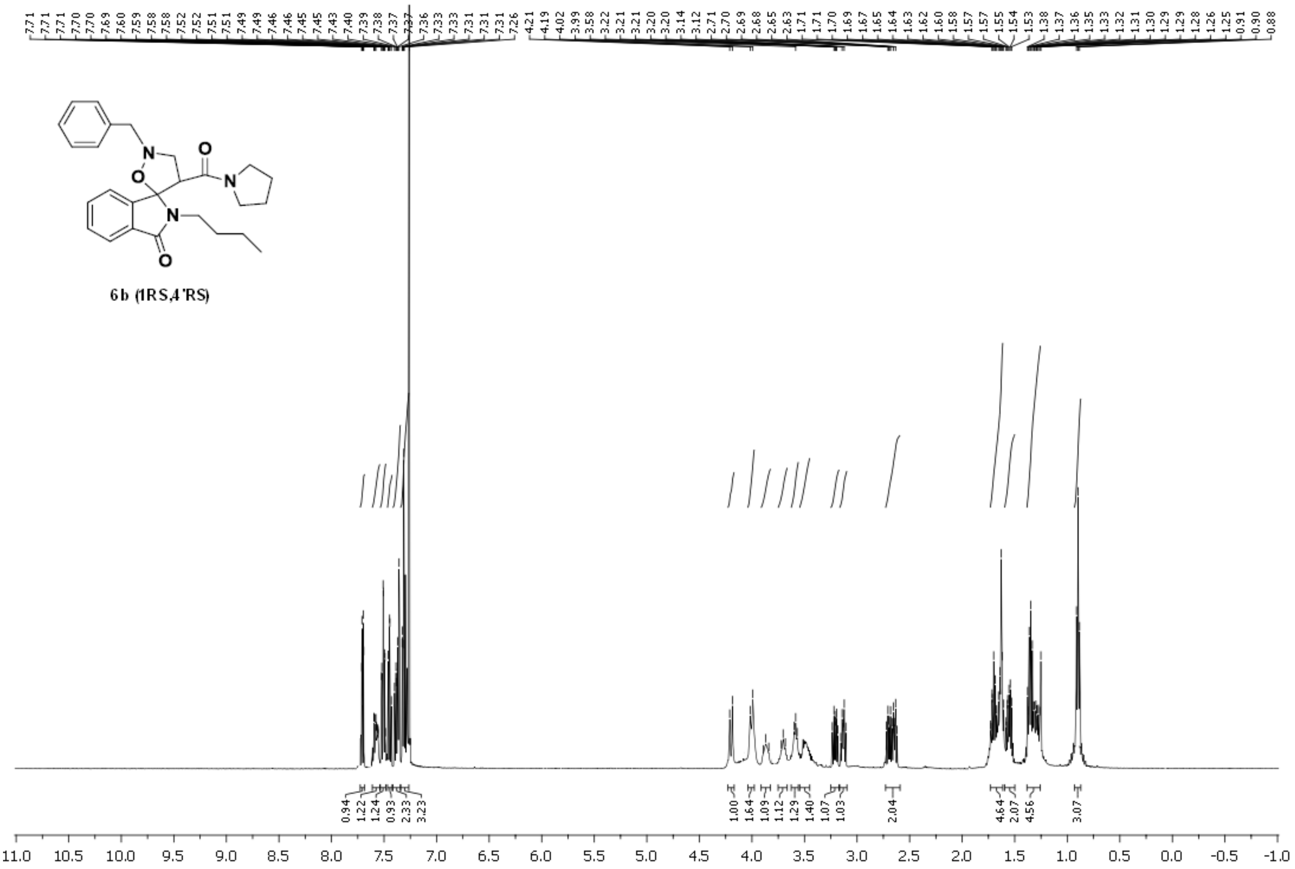

^{13}C NMR (125 MHz, $CDCl_3$) of compound **6b**

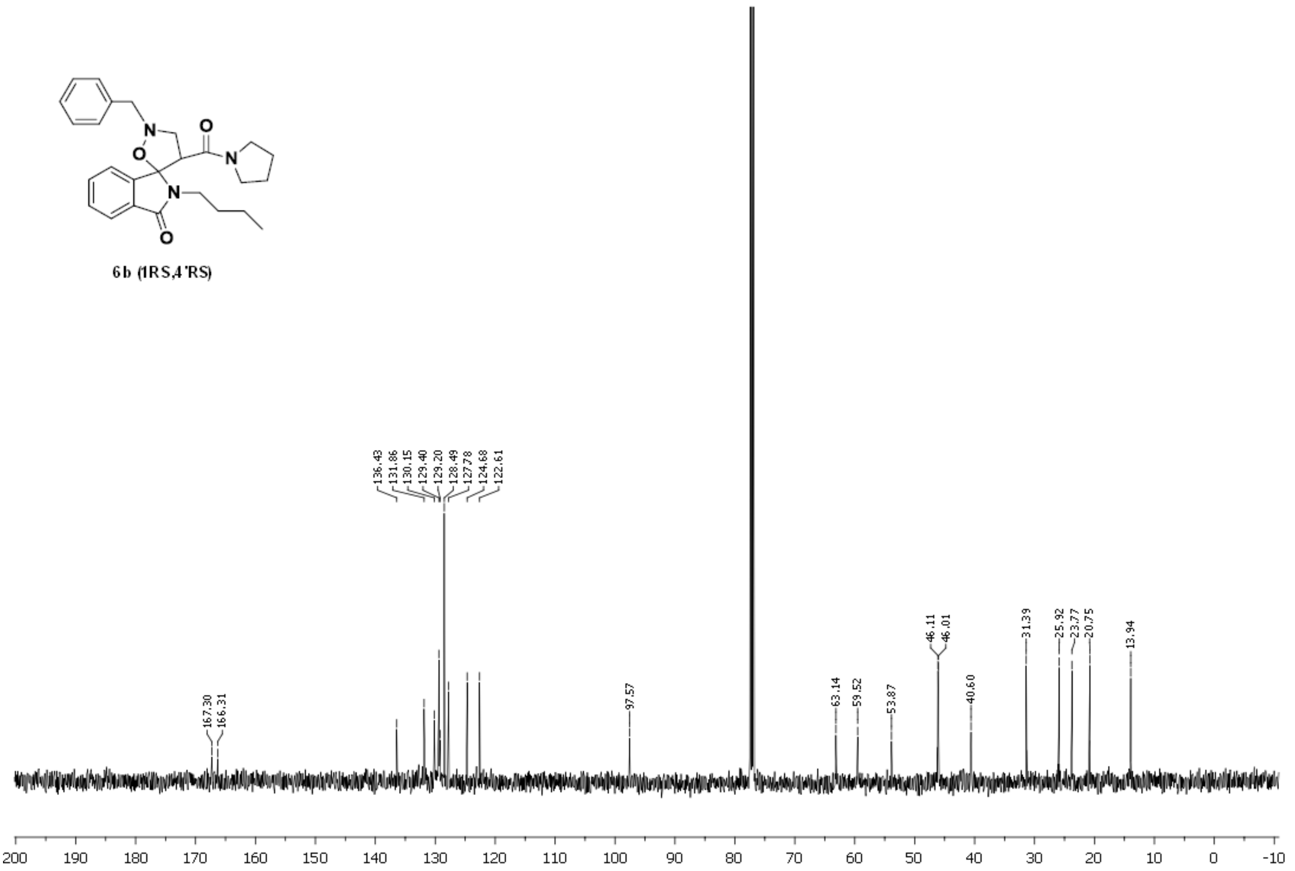

^{1}H NMR (500 MHz, $CDCl_3$) of compound **6c**

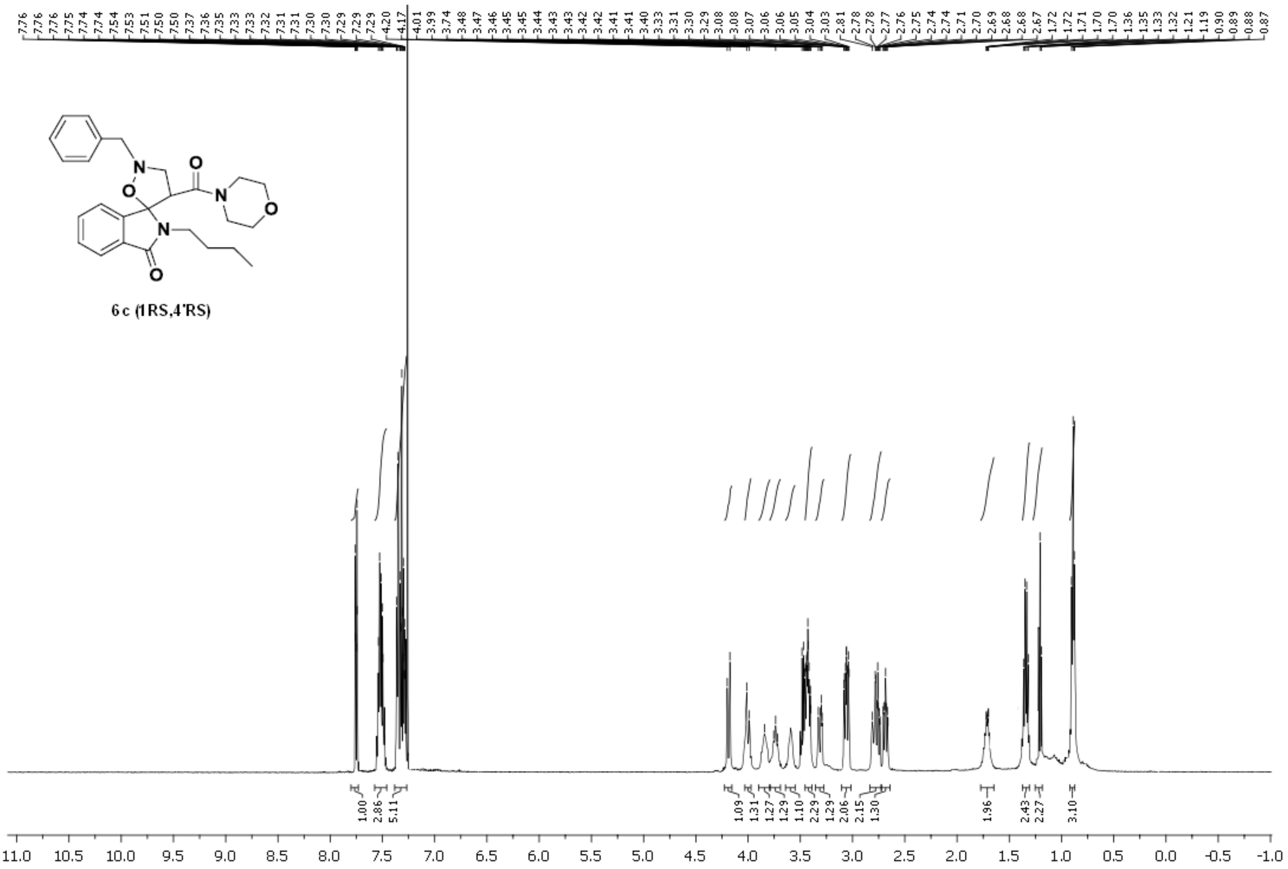

^{13}C NMR (125 MHz, $CDCl_3$) of compound **6c**

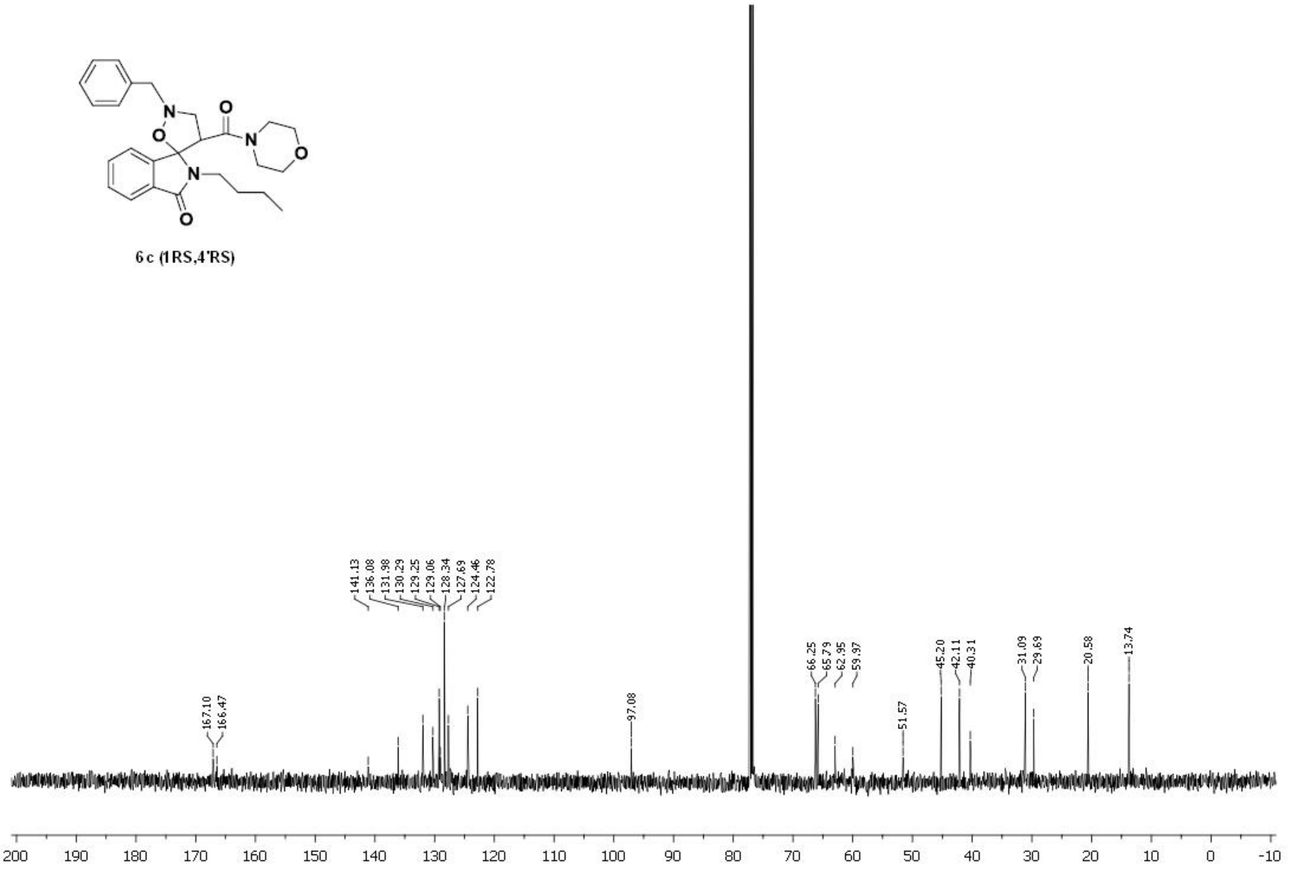

^{1}H NMR (500 MHz, $CDCl_3$) of compound **6d**

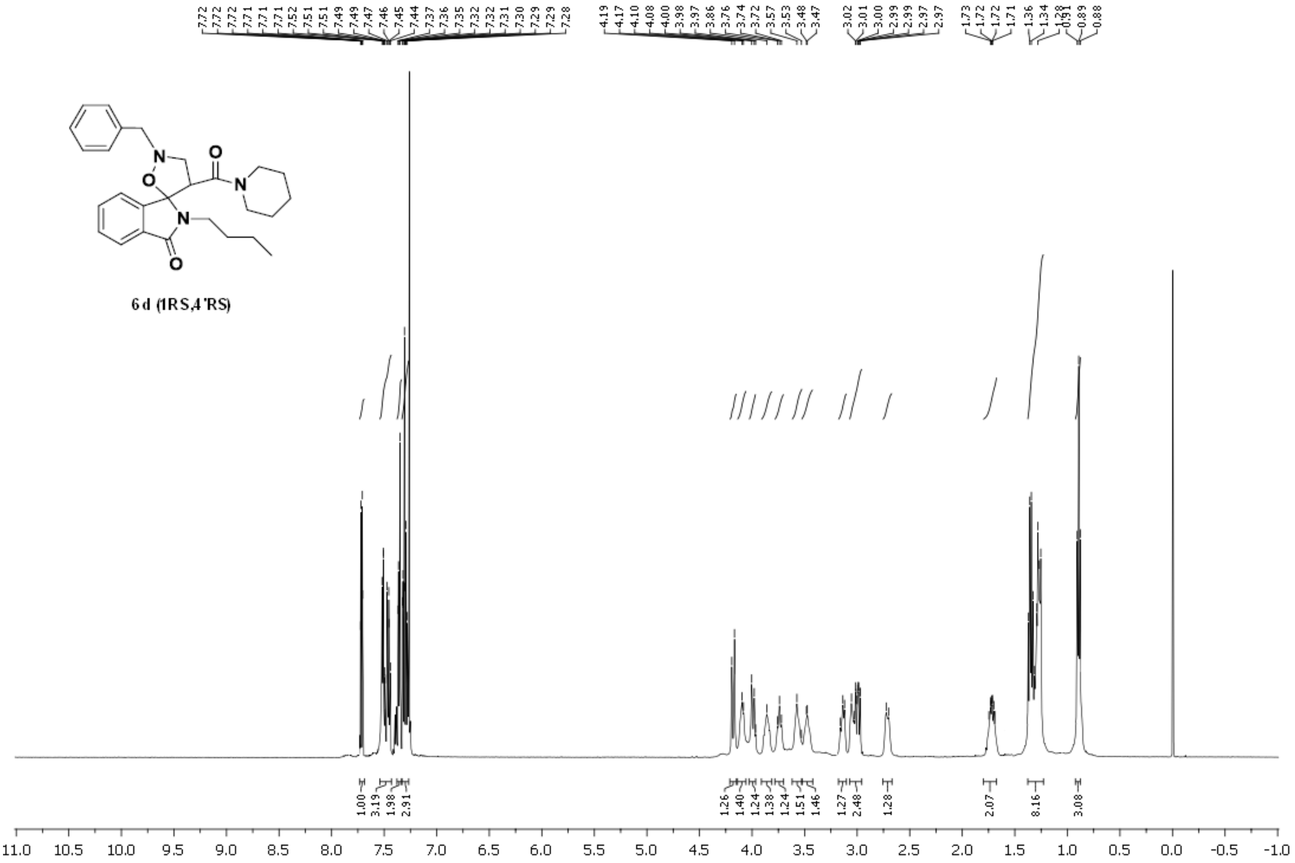

^{13}C NMR (125 MHz, $CDCl_3$) of compound **6d**

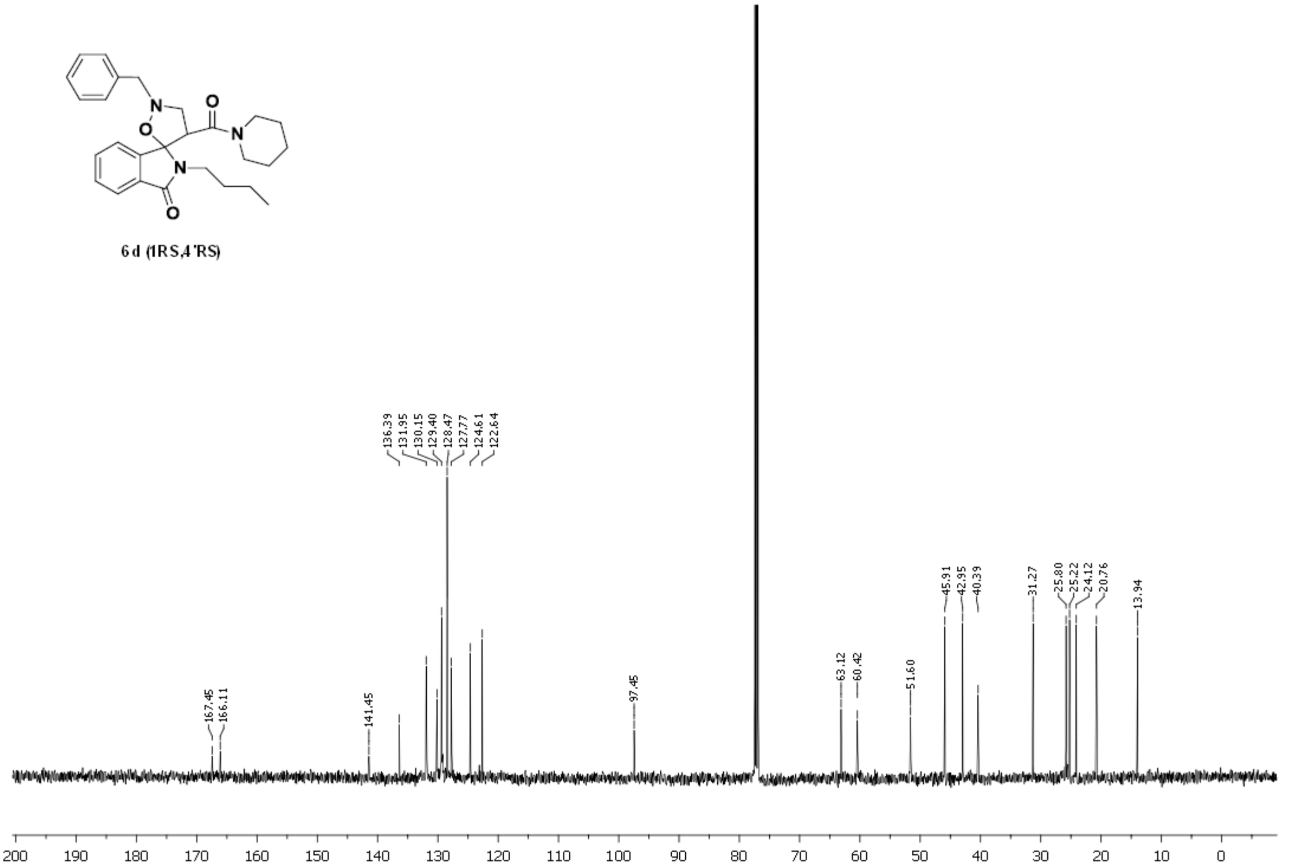

^{1}H NMR (500 MHz, $CDCl_3$) of compound **6e**

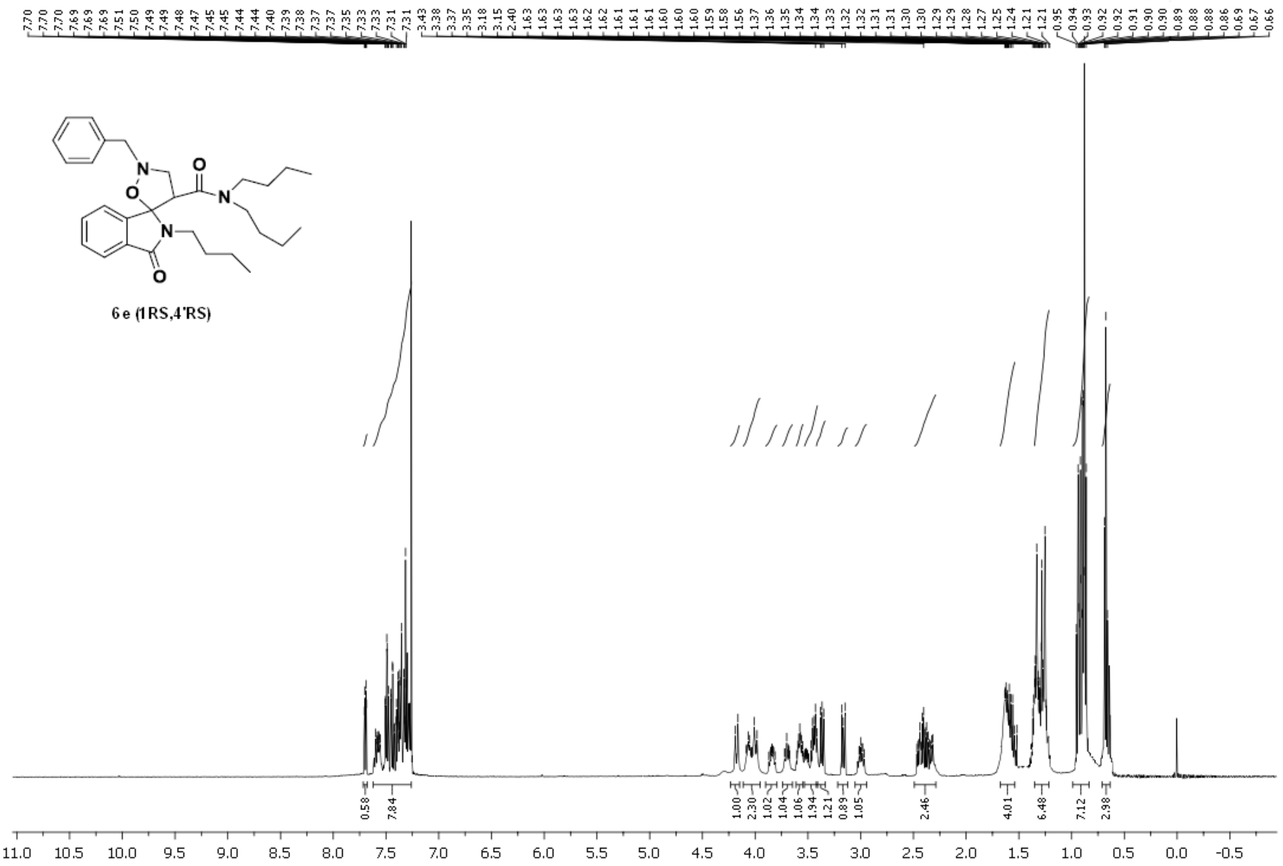

^{13}C NMR (125 MHz, $CDCl_3$) of compound **6e**

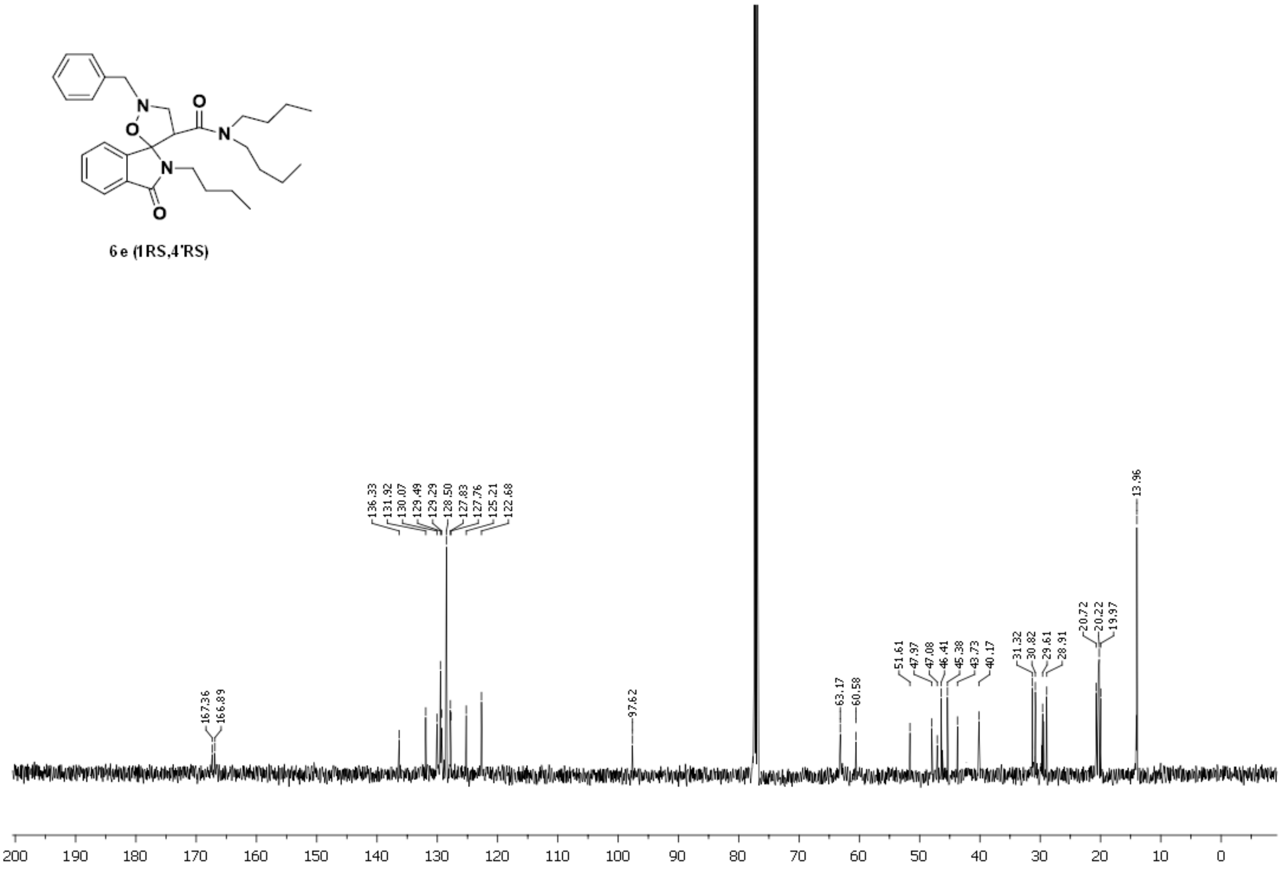

^{1}H NMR (500 MHz, $CDCl_3$) of compound **6f**

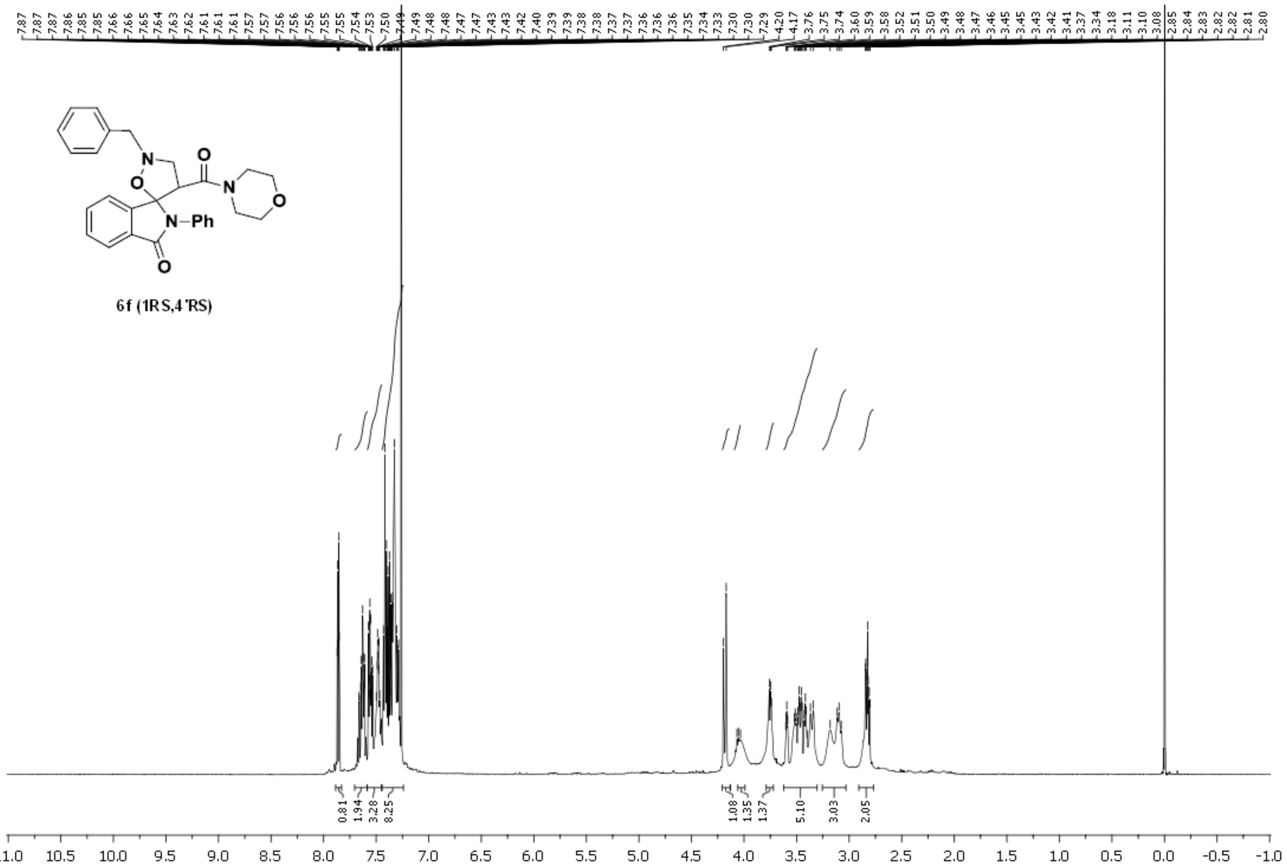

^{13}C NMR (125 MHz, $CDCl_3$) of compound **6f**

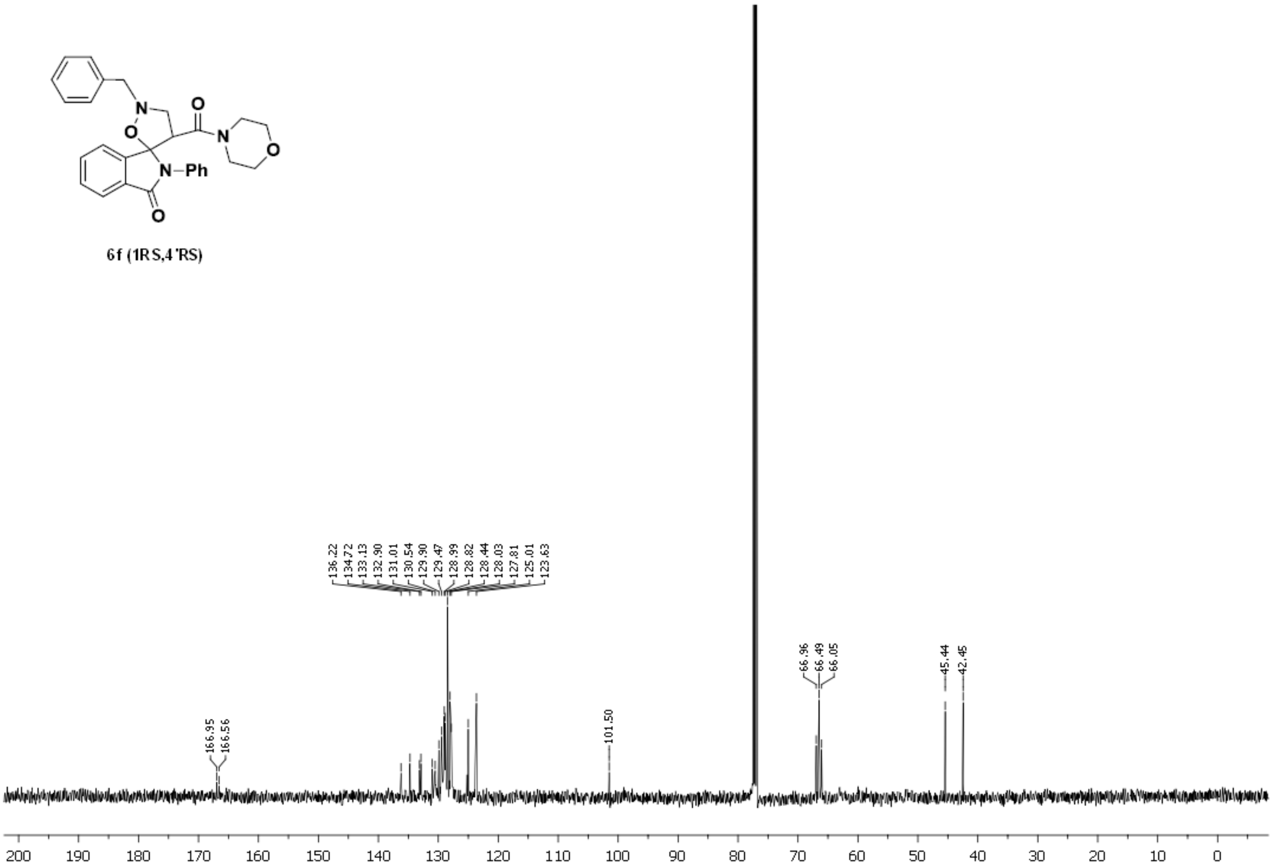

^{1}H NMR (500 MHz, $CDCl_3$) of compound **7a**

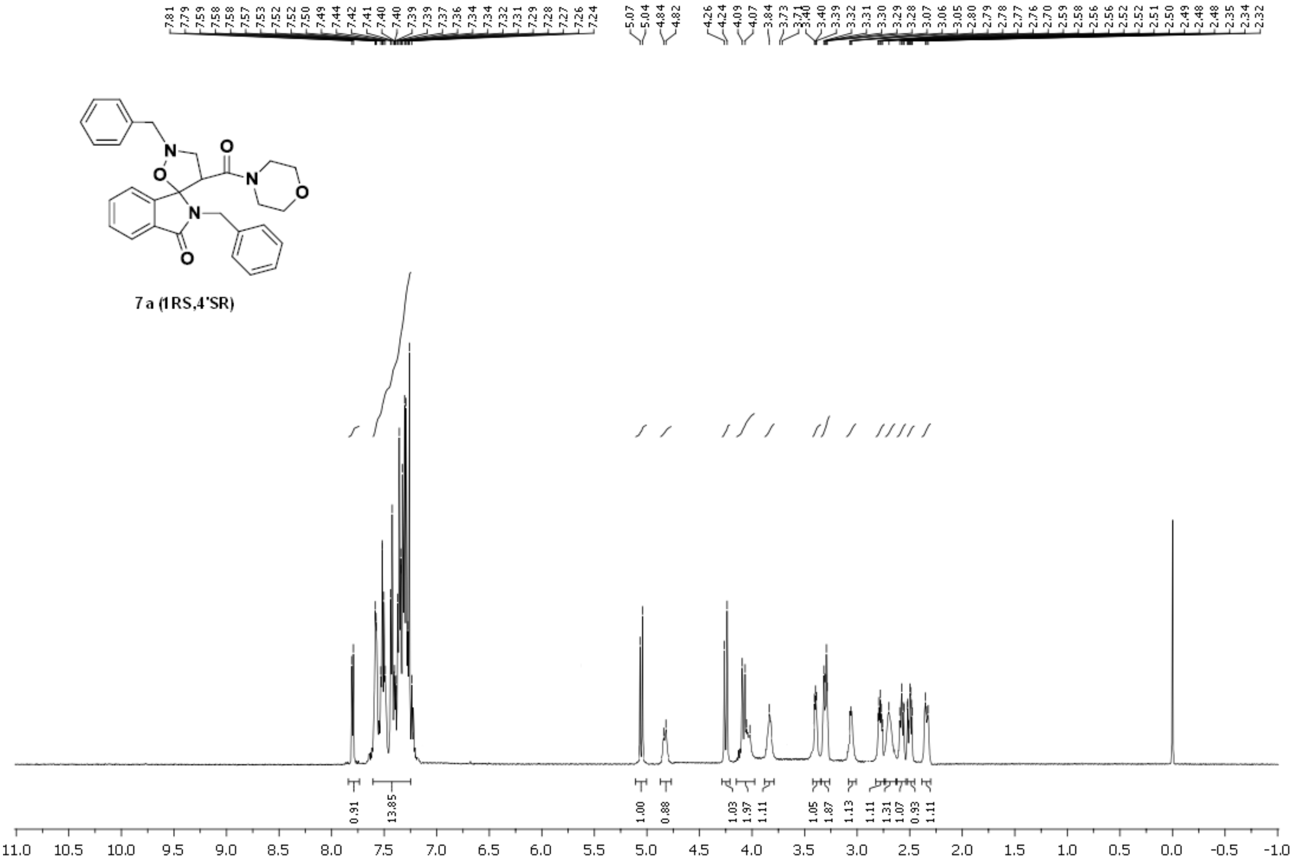

^{13}C NMR (125 MHz, $CDCl_3$) of compound **7a**

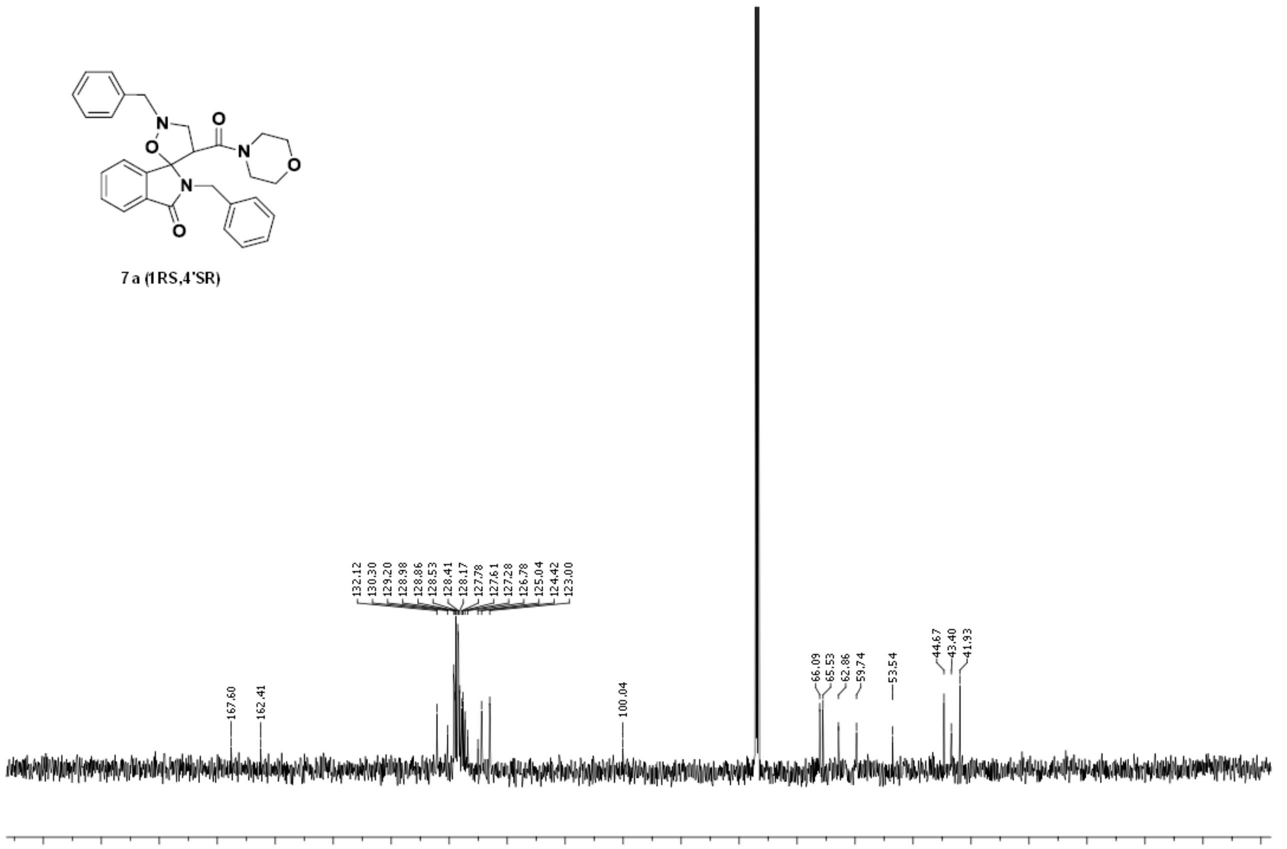

^1H NMR (500 MHz, $CDCl_3$) of compound **7g**

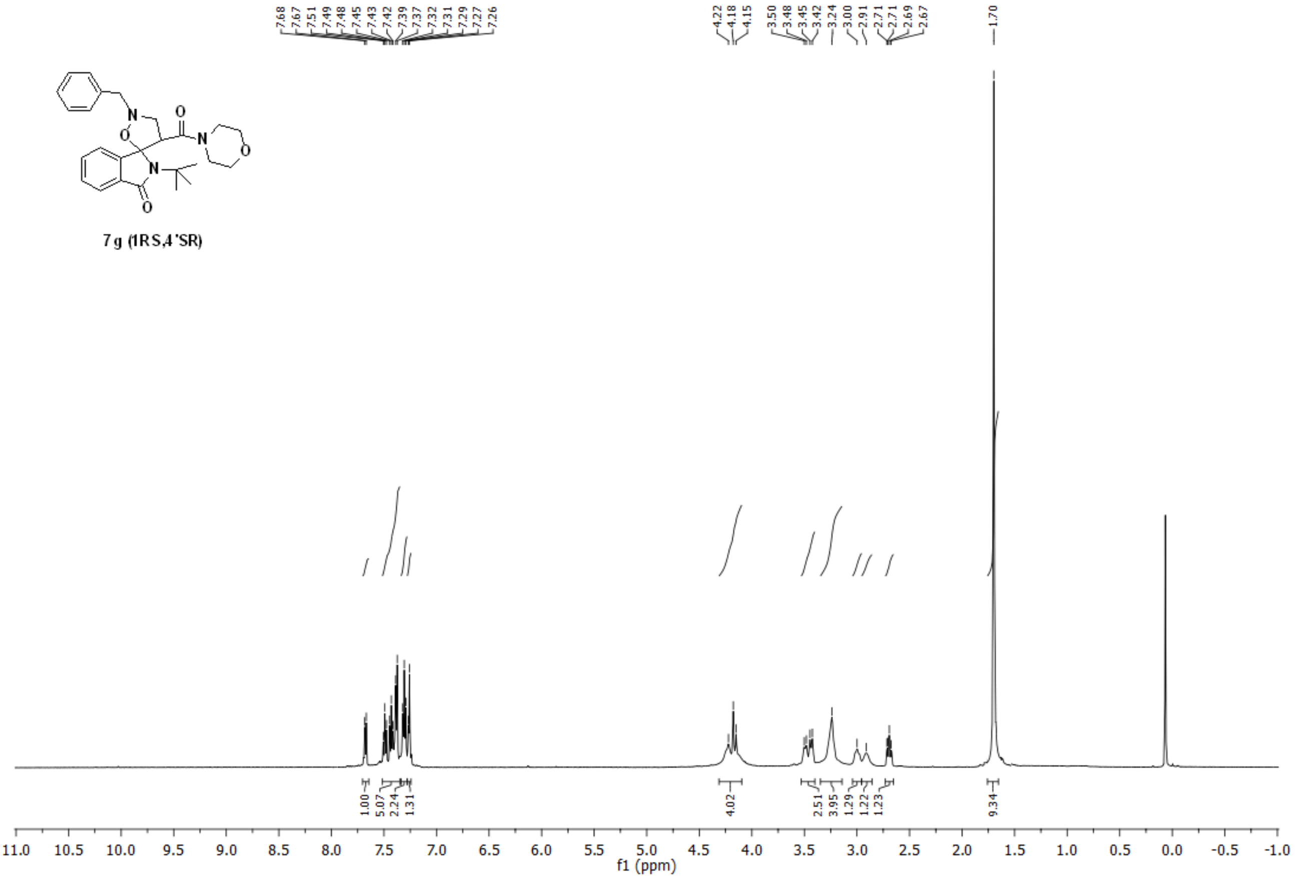

7g (1RS,4'SR)

^{13}C NMR (125 MHz, $CDCl_3$) of compound **7g**

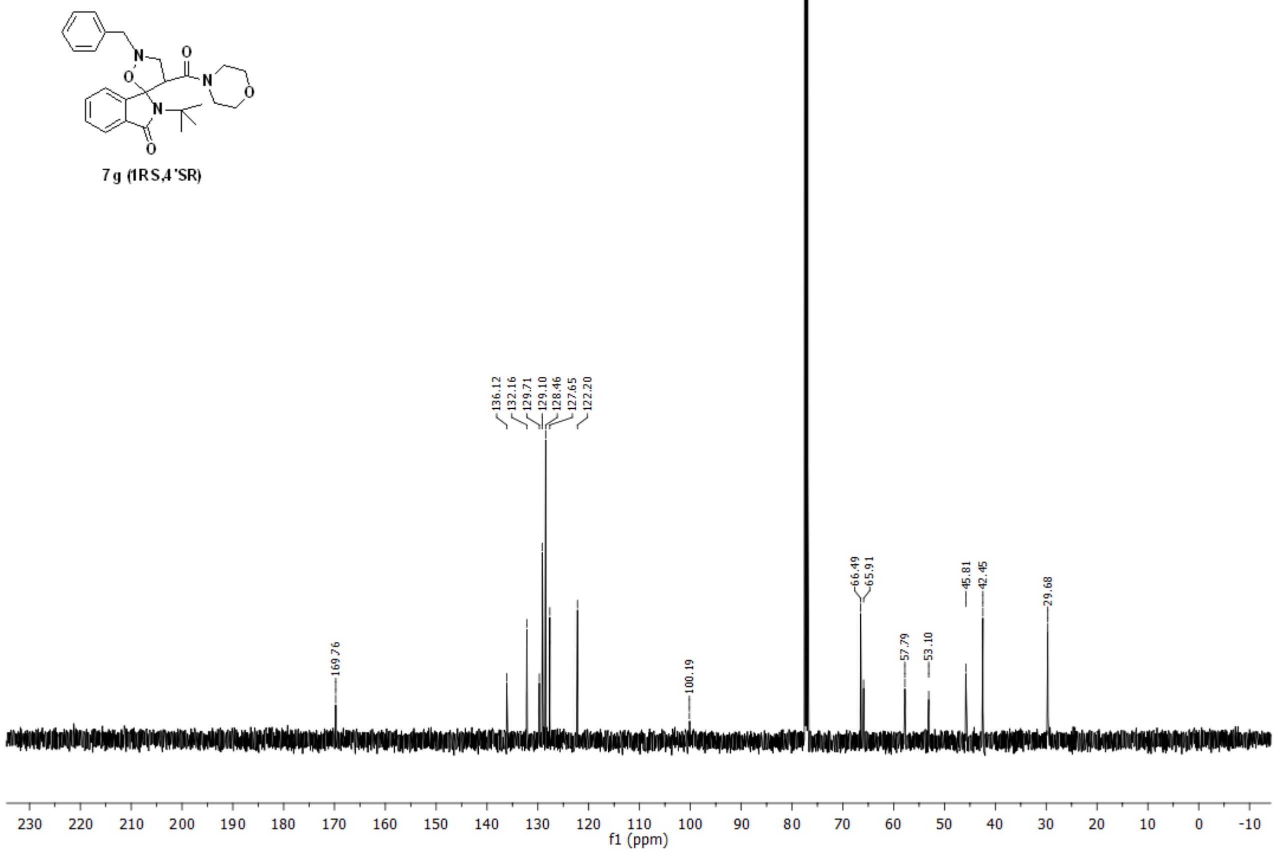

7g (1RS,4'SR)

Computational methods

All the calculations were carried out using the GAUSSIAN09 program package. All the structures of reactants, transition states, and products were optimized in the gas phase at the M06/6-31+G(d,p) level. The reaction pathways were confirmed by IRC analyses performed at the same level as above. Vibrational frequencies were computed at the same level of theory to define the optimized structures as minima or transition states, which present an imaginary frequency corresponding to the forming bonds. Thermodynamics at 298.15 K allowed the enthalpies and the Gibbs free energies to be calculated. The percentage contributions were determined through the Boltzmann equation.

***Z*-route**

4

G = -439.757229

C	-2.77407800	0.93098600	-0.64966300
H	-2.60128200	1.93288300	-0.27726000
H	-3.44470100	0.71312800	-1.47195500
N	-2.14630900	-0.06830300	-0.09927000
C	-1.17776000	0.19416500	1.01246100
O	-2.25208700	-1.27824600	-0.43947300
H	-1.38756600	1.18405700	1.43031700
H	-1.40489500	-0.57245500	1.75931000
C	0.23329000	0.09936800	0.51813500
C	0.83742400	-1.14467200	0.32572900
C	0.94902400	1.26123300	0.22651200
C	2.13925100	-1.22169500	-0.15754300
H	0.27024300	-2.04779800	0.54041100
C	2.25284600	1.18525600	-0.25364900
H	0.47987200	2.23366700	0.37790100
C	2.84909400	-0.05811500	-0.44603300
H	2.60234800	-2.19396300	-0.30823800
H	2.80376100	2.09603000	-0.47607200
H	3.86853900	-0.12062300	-0.81943100

8-Z

G = -684.609323

H	-1.31475500	-2.05500800	-0.13913900
C	-1.55127300	-0.99558100	-0.04311500
C	-0.48026300	-0.16895100	-0.02569400
C	0.91291800	-0.65807900	-0.01329700
C	1.43657600	-1.94407100	0.01580800
C	1.76063900	0.44519800	0.00542600
C	2.82335700	-2.08347400	0.04955400
H	0.79682100	-2.82412400	0.01415900
C	3.13935200	0.32045900	0.04446600
C	3.66759400	-0.96856500	0.06460400
H	3.25829000	-3.07991100	0.06826800
H	3.77064400	1.20575300	0.05923500
H	4.74454200	-1.11337500	0.09384500
N	-0.40403400	1.21714200	-0.03362800
C	0.92835000	1.66225900	-0.03284600
O	1.27652200	2.82335300	-0.07676500
C	-2.97962900	-0.68204000	0.13788400
O	-3.41377500	0.27842500	0.76109500
N	-3.82355300	-1.62471400	-0.39072400
H	-4.81500200	-1.45123300	-0.31408900
H	-3.50439400	-2.32436200	-1.04224600
C	-1.48854000	2.15613300	-0.25196200
H	-2.06250200	1.86575500	-1.13889000
H	-2.16609000	2.18059600	0.60227700
H	-1.03091700	3.13496900	-0.41256000

TS_ZN

G = -1124.32827

C	0.30926800	-2.48193200	-0.18143300
H	0.12685000	-3.34298200	0.46085700
H	0.94995800	-2.62490000	-1.04743900
N	-0.67236000	-1.58687500	-0.30143900
C	-1.70569400	-1.47361400	0.75331900
O	-0.40014000	-0.51157000	-0.94772600
H	-1.86731200	-2.47487800	1.16901800
H	-1.28108300	-0.82940200	1.53772300
H	1.29862000	-1.18447000	1.73675500
C	1.72553700	-1.25253100	0.73296400
C	1.62661900	-0.02460100	0.05578100
C	1.07964300	1.17894000	0.70365000
C	0.46043000	1.37560300	1.93515600
C	1.32163900	2.26639800	-0.13176000
C	0.05505000	2.66448300	2.27060800
H	0.28259800	0.55357200	2.62768100
C	0.92494800	3.55484100	0.19318400
C	0.27473700	3.74628700	1.40854800
H	-0.44017900	2.83493700	3.22394900
H	1.12779900	4.37655300	-0.48969500
H	-0.05564400	4.73991100	1.70043100
N	2.23855000	0.40356500	-1.11462200
C	2.03170300	1.77898100	-1.32260000
O	2.38837300	2.39599100	-2.30684400
C	2.84967900	-2.21937400	0.65249700
O	3.37496600	-2.60891800	-0.38051000
N	3.21364500	-2.74234600	1.86918600
H	4.02908100	-3.33928900	1.86568000
H	2.98520800	-2.27160500	2.73135000
C	-2.98484700	-0.89247700	0.23627400
C	-3.11736400	0.48809400	0.07148500
C	-4.05537400	-1.72606500	-0.08614000
C	-4.30663800	1.02304000	-0.41135300
H	-2.27425600	1.13586800	0.30850200
C	-5.24721600	-1.19188500	-0.56762200
H	-3.95346400	-2.80363400	0.04105900
C	-5.37315000	0.18498700	-0.72993600
H	-4.40137100	2.09847400	-0.54105900
H	-6.07701200	-1.84992300	-0.81437000
H	-6.30343200	0.60570400	-1.10467300
C	2.66558100	-0.41929800	-2.23603400

H	3.53132800	-1.02664300	-1.97791200
H	1.84257800	-1.07025300	-2.55007100
H	2.90798100	0.26926200	-3.04854600

TS_ZX
G = -1124.329707

C	0.91236700	-0.88146300	0.94578200
H	0.31193800	-1.62322900	1.46677300
H	1.86887300	-0.60576300	1.38992600
N	0.86499900	-0.97605100	-0.38857300
C	1.85250700	-0.31087400	-1.26972100
O	-0.29895300	-1.19450500	-0.89022900
H	1.57999300	0.75155000	-1.33993700
H	1.69645600	-0.78233000	-2.24599200
H	-0.75419300	0.22510200	2.23856100
C	-0.42554900	0.61113000	1.27125100
C	-1.43625500	0.45551100	0.29693000
C	-2.57413400	-0.43429400	0.54324100
C	-2.79218200	-1.40466400	1.51505900
C	-3.53837100	-0.19451000	-0.43289500
C	-3.98891300	-2.11751500	1.47288400
H	-2.05822200	-1.61651300	2.29127700
C	-4.73125400	-0.89669600	-0.48393100
C	-4.95043500	-1.87091000	0.48694000
H	-4.17812800	-2.88367900	2.22132000
H	-5.46007100	-0.68275300	-1.26227600
H	-5.87258600	-2.44653900	0.48294200
N	-1.79510900	1.25512200	-0.78707300
C	-3.04074100	0.87240200	-1.31247200
O	-3.56271400	1.36564800	-2.29398900
C	0.47023400	1.78841900	1.40488300
O	1.18390000	2.22731100	0.50973300
N	0.52344700	2.31527100	2.66632300
H	1.08843800	3.14357700	2.79021800
H	-0.18148600	2.11229700	3.35790700
C	3.26256100	-0.47717700	-0.79038600
C	3.94823300	0.58858100	-0.20530100
C	3.89898100	-1.71599900	-0.91066400
C	5.25288200	0.41735300	0.25304300
H	3.44295500	1.54812900	-0.10178600
C	5.19914400	-1.88881800	-0.44983000
H	3.36390800	-2.54765600	-1.36892700
C	5.87869900	-0.82025700	0.13339000
H	5.78090100	1.25369600	0.70548000
H	5.68604200	-2.85628600	-0.54928500
H	6.89690400	-0.95352000	0.49185400
C	-0.96966500	2.17874400	-1.53352100
H	-0.56126200	2.96251000	-0.89548400
H	-0.14006500	1.64555900	-2.01554100
H	-1.61027700	2.61403200	-2.30399000

9_N
G = -1124.384797

C	-0.10017400	-2.46968100	-0.64251200
H	0.22813800	-3.07278800	-1.49477900
H	-0.57131200	-3.13923000	0.08449300
N	1.04354200	-1.78946200	-0.04123500
C	2.01107200	-1.30534700	-1.02485800
O	0.48063900	-0.64777900	0.62065000
H	2.39331900	-2.21052600	-1.51983400
H	1.54544500	-0.67789400	-1.80950900
H	-0.71516800	-0.86051700	-1.95110800
C	-1.05051100	-1.34540000	-1.02296400
C	-0.83328000	-0.35013100	0.13748400
C	-0.97327600	1.10348700	-0.24288700
C	-0.32177700	1.82577300	-1.23086100
C	-1.91127600	1.71182800	0.57806400
C	-0.64461900	3.17628300	-1.37381900
H	0.43327900	1.36561900	-1.86786500
C	-2.24122600	3.05185400	0.44775800
C	-1.59374900	3.78329100	-0.54634200
H	-0.14449400	3.76818900	-2.13666600
H	-2.97825800	3.50062400	1.10967100
H	-1.82211500	4.83779900	-0.68009200
N	-1.79126900	-0.47674300	1.22641500
C	-2.41786000	0.70970300	1.54814100
O	-3.21898100	0.88153600	2.44758900
C	-2.49783300	-1.76446700	-1.20215800
O	-2.90386000	-2.87280100	-0.89583900
N	-3.29792300	-0.81233500	-1.75395600
H	-4.29053800	-0.99873200	-1.78468900
H	-2.98357900	0.14021900	-1.87780500
C	-1.88586800	-1.65053500	2.06878900
H	-2.40829700	-2.47046400	1.56223000
H	-0.88222800	-1.97474600	2.36733100
H	-2.45397700	-1.36638100	2.95834000
C	3.14270100	-0.55113300	-0.38656800
C	3.57035300	0.66208900	-0.92159700
C	3.79882800	-1.06794300	0.73256500
C	4.64333400	1.34968200	-0.35822200
H	3.05536000	1.07666300	-1.78872200
C	4.86702900	-0.38282800	1.29919200
H	3.45134500	-2.00500800	1.16323000
C	5.29428700	0.82737100	0.75416900
H	4.96549100	2.29647200	-0.78569100
H	5.36870300	-0.79295700	2.17277100
H	6.12913700	1.36240400	1.20069100

9_X
G = -1124.383166

C	-0.86199500	0.70281400	1.12718900
H	-0.63778500	1.65736600	1.61884400
H	-1.76211400	0.27473700	1.57923400
N	-1.08886700	0.95233600	-0.29134000
C	-1.96500400	-0.02357100	-0.95890600
O	0.21569400	0.90153800	-0.87018800
H	-1.70316900	-1.06778900	-0.72809500
H	-1.82263500	0.14637700	-2.03358800
H	0.99012800	0.07516000	2.05803200
C	0.34793400	-0.22404000	1.21951100
C	1.11406100	0.09762900	-0.10374400
C	2.38789400	0.87237200	0.15209200
C	2.55386800	2.09534000	0.78246400
C	3.47483600	0.18102300	-0.35685700
C	3.85140100	2.59482500	0.89820900
H	1.70229000	2.66281200	1.15441500

C	4.77049400	0.66498100	-0.24710900
C	4.94864400	1.88725300	0.39581600
H	4.01292700	3.55685200	1.37900600
H	5.60328500	0.10322400	-0.66370400
H	5.94676500	2.30527800	0.50073500
N	1.61010700	-1.04937100	-0.85671500
C	2.98186600	-1.03532400	-1.04030300
O	3.62879700	-1.86773700	-1.64921200
C	0.01096200	-1.69247400	1.44459700
O	-1.11670200	-2.14938600	1.33580200
N	1.06766400	-2.45466800	1.83305800
H	0.92206800	-3.45159000	1.91052300
H	2.01705500	-2.11679700	1.76468900
C	0.77351200	-1.96674300	-1.59656200
H	0.15724700	-2.59111500	-0.93963600
H	0.12232800	-1.41828900	-2.28629600
H	1.44125300	-2.61388700	-2.17121700
C	-3.39492000	0.23691600	-0.57548300
C	-4.12454600	-0.71097200	0.14237100
C	-4.00914500	1.43904600	-0.93740500
C	-5.45448200	-0.46701600	0.48185500
H	-3.63913100	-1.63905900	0.44183800
C	-5.33445400	1.68469500	-0.59772000
H	-3.43319600	2.18301000	-1.48595700
C	-6.06143900	0.72883200	0.11140200
H	-6.01517300	-1.21400300	1.03926200
H	-5.80433500	2.62176100	-0.88820800
H	-7.09937400	0.91903200	0.37536400

***E*-route**

8-E

G = -684.615953

H	2.37375900	1.52137700	0.00087900
C	1.91136900	0.53552000	0.00039900
C	0.55574700	0.51050800	0.00016500
C	-0.45775900	-0.56882600	-0.00005000
C	-0.37383000	-1.95887300	0.00005700
C	-1.71803700	0.04266100	-0.00015400
C	-1.56544200	-2.68734100	0.00008900
H	0.59280200	-2.45089800	0.00012900
C	-2.90105900	-0.67287000	-0.00011900
C	-2.81382300	-2.06349100	0.00000400
H	-1.51342400	-3.77354700	0.00020300
H	-3.85510500	-0.15090900	-0.00017700
H	-3.71851900	-2.66650900	0.00005200
N	-0.16601400	1.70838900	0.00017700
C	-1.54754100	1.50351700	-0.00007200
O	-2.38518300	2.38301800	-0.00018900
C	2.84335000	-0.59772200	0.00002000
O	2.53527600	-1.78464900	-0.00004600
N	4.16207200	-0.22544300	0.00015700
H	4.85180800	-0.96157700	-0.00169900
H	4.46888300	0.73367000	-0.00229300
C	0.40389000	3.03098500	0.00005800
H	1.01619500	3.19835900	0.89452600
H	1.01663000	3.19798000	-0.89418500
H	-0.42736600	3.74008600	-0.00027200

TS_EN

G = -1124.333474

C	0.27667900	2.36439700	-0.91000800
H	0.63525700	3.27677700	-0.43365200
H	-0.11526600	2.45233500	-1.91913000
N	0.95274200	1.23682400	-0.65719100
C	1.85993000	1.09729800	0.50430700
O	0.42153200	0.15601100	-1.10486900
H	2.15364900	2.10588400	0.81526600
H	1.27540800	0.64736900	1.31454900
H	-2.15764000	2.43394000	-0.83064300
C	-1.54967600	1.85609900	-0.13177100
C	-1.66607700	0.46084000	-0.31787900
C	-1.57524200	-0.67766200	0.61761800
C	-1.09505100	-0.81462900	1.92007700
C	-2.11903500	-1.79333400	-0.03039500
C	-1.16612600	-2.07028100	2.51956000
H	-0.67593700	0.03257300	2.45316400
C	-2.18960100	-3.04496500	0.56088500
C	-1.69993500	-3.17964100	1.85542800
H	-0.79214500	-2.18765300	3.53445100
H	-2.62027100	-3.88023700	0.01340600
H	-1.73445900	-4.14390400	2.35647000
N	-2.31651200	-0.02790400	-1.45201200
C	-2.57467400	-1.39796600	-1.36845500
O	-3.08388600	-2.06756400	-2.24810500
C	-1.41411200	2.49439800	1.19709900
O	-0.62813600	2.13332200	2.06598100
N	-2.17055500	3.62537900	1.36175600
H	-2.14313000	4.05426500	2.27626800
H	-2.96868400	3.81673500	0.77608100
C	3.05286700	0.26109600	0.15358100
C	3.00037000	-1.12753400	0.28200300
C	4.22174500	0.85970100	-0.31745700
C	4.10296500	-1.90567000	-0.05586200
H	2.08229700	-1.59270600	0.63856000
C	5.32661700	0.08397100	-0.65524100
H	4.26491500	1.94434300	-0.41753900
C	5.26753600	-1.30117700	-0.52376300
H	4.05310800	-2.98722900	0.04542500
H	6.23495300	0.55988600	-1.01768400
H	6.13048600	-1.90973500	-0.78438700
C	-2.52551200	0.69680600	-2.67872700
H	-3.21544800	1.53911200	-2.54087800
H	-1.57439100	1.05712700	-3.08724900
H	-2.97051000	-0.00360800	-3.39040800

TS_EX

G = -1124.329479

C	-1.05218800	1.03477900	-0.64588600
H	-0.42635800	1.79799400	-1.10305600
H	-2.01568000	1.34240700	-0.24653800
N	-0.94998500	-0.16995500	-1.21360800
C	-1.89261000	-1.26698000	-0.90938700
O	0.21604400	-0.54064500	-1.59579300
H	-1.46337400	-1.82981800	-0.06546400
H	-1.84596900	-1.92214200	-1.78634300
H	-0.56425300	0.28596700	1.64390600

C	0.14556300	0.80154200	0.99358500
C	1.25102500	0.02735200	0.61336600
C	2.44159600	0.34246000	-0.17989900
C	2.76732700	1.41387200	-1.01157200
C	3.31498800	-0.74835600	-0.09145400
C	3.98196500	1.37677600	-1.69235400
H	2.09148800	2.25405200	-1.14804500
C	4.52202700	-0.79315800	-0.76951400
C	4.85941000	0.29420300	-1.57009700
H	4.24758000	2.20636000	-2.34355600
H	5.16644500	-1.66423500	-0.67636100
H	5.79817300	0.29865900	-2.11800000
N	1.45844500	-1.25384900	1.12865700
C	2.69937200	-1.78075200	0.75282500
O	3.13076600	-2.86584400	1.09616300
C	0.13643600	2.24972000	1.36903700
O	-0.90123800	2.77701400	1.74699200
N	1.29672700	2.95906600	1.26294200
H	1.26939100	3.90225500	1.62466400
H	2.19509800	2.50462100	1.18338300
C	-3.28790000	-0.81715000	-0.61270600
C	-3.76248300	-0.78376200	0.69897000
C	-4.12703500	-0.40261800	-1.65041500
C	-5.05348200	-0.34076700	0.97380200
H	-3.11203700	-1.10610100	1.51230800
C	-5.41590500	0.04237100	-1.37930900
H	-3.75907000	-0.42799900	-2.67582200
C	-5.88087200	0.07355500	-0.06569800
H	-5.41135200	-0.31719600	2.00003400
H	-6.06122100	0.36254300	-2.19377200
H	-6.88929500	0.42027900	0.14657900
C	0.59356500	-1.95190700	2.04104700
H	0.41191700	-1.36446000	2.94917300
H	-0.37217900	-2.19627300	1.57654600
H	1.09501200	-2.88502700	2.30947500

10_N

G = -1124.382767

C	-0.01011000	-2.70595100	-0.21966700
H	0.34958500	-3.24749400	-1.09946000
H	-0.26875800	-3.42434600	0.56832400
N	1.03035000	-1.81830000	0.28218600
C	1.90639500	-1.25145300	-0.74232900
O	0.32337600	-0.77619700	0.94296100
H	2.39092100	-2.12246600	-1.20870400
H	1.35107900	-0.74799000	-1.54958000
H	-2.14821000	-2.32197600	-0.19690400
C	-1.22512000	-1.82696200	-0.52465400
C	-0.99603200	-0.60024500	0.40842900
C	-1.19047300	0.78427000	-0.17736600
C	-0.54992600	1.41229700	-1.23585000
C	-2.15636200	1.46169700	0.55697400
C	-0.92208900	2.72136000	-1.54296600
H	0.21535000	0.90812600	-1.82262600
C	-2.53450000	2.76228500	0.26223000
C	-1.90459200	3.39104500	-0.80874000
H	-0.43181700	3.23174400	-2.36874800
H	-3.29164300	3.26047200	0.86336600
H	-2.16834300	4.41229500	-1.07244300
N	-1.93475300	-0.59263700	1.51713000
C	-2.62496400	0.59330800	1.66068700
O	-3.44660800	0.84391300	2.52297300
C	-1.39936000	-1.48182300	-1.99527800
O	-0.49765600	-1.55834400	-2.81485000
N	-2.66373700	-1.11086700	-2.33646500
H	-2.80315100	-0.74523800	-3.26830300
H	-3.35687600	-0.89213700	-1.63506700
C	-2.00035200	-1.66294600	2.47854500
H	-2.43891600	-2.57483900	2.04919700
H	-0.99876500	-1.89147300	2.85977800
H	-2.63816600	-1.32875200	3.30093400
C	2.94758200	-0.33346500	-0.16567500
C	3.38429200	0.76350300	-0.90839700
C	3.52492300	-0.57598500	1.08219300
C	4.38536300	1.60095100	-0.42245100
H	2.93599300	0.96148400	-1.88242400
C	4.52054700	0.26163100	1.57246300
H	3.17344000	-1.41936700	1.67229700
C	4.95609400	1.35211900	0.82175000
H	4.71333400	2.45241600	-1.01466900
H	4.95816300	0.06550900	2.54891500
H	5.73296700	2.00787600	1.20809300

10_X

G = -1124.387456

C	0.97228400	-1.00151100	0.36227500
H	0.61832600	-2.00838300	0.11375300
H	1.88692700	-1.10434700	0.95477100
N	1.24831800	-0.25220600	-0.86010800
C	2.15967200	0.88325600	-0.67426000
O	-0.03124900	0.25976800	-1.25421500
H	1.86434800	1.54309900	0.16251500
H	2.08672800	1.47513900	-1.59573600
H	0.30516700	0.66595700	1.56481700
C	-0.12182400	-0.20960600	1.05331900
C	-0.94767200	0.30249000	-0.14834400
C	-2.18528300	-0.49266900	-0.50498000
C	-2.31240900	-1.83517600	-0.82369700
C	-3.29048900	0.34643400	-0.52960800
C	-3.58494200	-2.30979300	-1.14507500
H	-1.45514500	-2.50377800	-0.83351200
C	-4.55844200	-0.11032100	-0.85265700
C	-4.69598800	-1.46220400	-1.15806500
H	-3.71286800	-3.35998800	-1.39615300
H	-5.40165600	0.57609900	-0.86802700
H	-5.67224200	-1.86424500	-1.41738700
N	-1.49584700	1.63478800	0.05596400
C	-2.85665700	1.71967200	-0.19861500
O	-3.52710700	2.73314600	-0.13320600
C	-0.91329700	-0.99394900	2.08218800
O	-0.72434100	-2.17879700	2.29676000
N	-1.82272000	-0.25166400	2.77207800
H	-2.44801600	-0.73891400	3.39836800
H	-2.05875400	0.68957600	2.48879200
C	-0.68619100	2.81102800	0.24936800
H	-0.16295300	2.79690600	1.21557800

H	0.05161300	2.91352200	-0.55522800
H	-1.35688000	3.67413300	0.23400200
C	3.56550600	0.39707900	-0.46411000
C	4.17882200	-0.42195800	-1.41622400
C	4.27574300	0.75228800	0.68233700
C	5.48131100	-0.86812900	-1.22545900
H	3.61774500	-0.71094800	-2.30336000
C	5.58335100	0.31061400	0.87374700
H	3.79919200	1.38332500	1.43303300
C	6.18780300	-0.50076200	-0.08069400
H	5.94975400	-1.50469200	-1.97269200
H	6.12694000	0.59725100	1.77115600
H	7.20730100	-0.84978000	0.06637400

6a

C	-1.47361600	-0.61784600	-1.06722100
H	-2.08822500	-1.46197100	-1.39802500
H	-1.39974600	0.09596500	-1.89334800
N	-2.08412300	0.01639900	0.09095300
C	-2.67276800	-0.93029800	1.04327800
O	-1.00931900	0.70297800	0.72199400
H	-2.01839900	-1.80029400	1.25061500
H	-2.78945100	-0.37629700	1.98321900
H	-0.13142800	-1.97190900	-0.04704100
C	-0.07439400	-1.02573700	-0.60057700
C	0.25848800	0.10937600	0.41274200
C	0.97506400	-0.35115900	1.66319400
C	0.59710500	-1.28786900	2.61422600
C	2.18440900	0.31571800	1.78002200
C	1.48008500	-1.55343600	3.66144100
H	-0.35919100	-1.80482800	2.55745400
C	3.07182200	0.06451300	2.81543500
C	2.70806300	-0.89155400	3.76082700
H	1.20536000	-2.28438500	4.41834700
H	4.01542400	0.60322800	2.87036400
H	3.37454700	-1.12051600	4.58856300
N	1.16355600	1.13279800	-0.09531100
C	2.30736000	1.27615500	0.66013900
O	3.22870900	2.04242900	0.43501800
C	0.89396700	-1.16871900	-1.77202300
O	0.61943000	-0.65617700	-2.85236100
C	0.86493700	2.04495000	-1.19857900
H	0.37837800	1.48701600	-2.00523500
H	1.84411400	2.36503100	-1.57723500
C	2.50770200	-2.55809200	-0.38853700
C	2.95906000	-2.04215800	-2.72088300
C	3.93368500	-2.12640400	-0.08034100
H	2.48706600	-3.64657700	-0.55861400
H	1.86747700	-2.32965300	0.46743300
C	4.34936900	-1.61106900	-2.30320700
H	2.97479400	-3.10185300	-3.01920400
H	2.58359700	-1.44549600	-3.55529400
H	4.33078200	-2.69997600	0.76424900
H	3.94586700	-1.05463600	0.18759400
H	5.07250200	-1.79193800	-3.10437600
H	4.34735000	-0.53172400	-2.06337700
N	2.04221200	-1.88385500	-1.59409300
O	4.78705000	-2.35042100	-1.17885600
C	-4.00213800	-1.41406900	0.53674100
C	-5.01946000	-0.49693900	0.25857600
C	-4.23970400	-2.77272000	0.33302900
C	-6.25421700	-0.93562900	-0.20418300
H	-4.82480100	0.56469700	0.40031400
C	-5.47737200	-3.21576000	-0.12959100
H	-3.44623300	-3.49169700	0.53878500
C	-6.48677900	-2.29711000	-0.39780800
H	-7.03988000	-0.21382800	-0.41523100
H	-5.65028500	-4.27842400	-0.28334700
H	-7.45320700	-2.63952000	-0.76034500
C	0.04503300	3.24468400	-0.79924400
C	0.56745300	4.19180300	0.08496800
C	-1.23433700	3.43447100	-1.31840800
C	-0.18467900	5.30325000	0.44999200
H	1.57438000	4.05492300	0.47791600
C	-1.98742900	4.54939500	-0.95886300

H	-1.64767100	2.69706900	-2.00657400
C	-1.46454900	5.48462000	-0.07104600
H	0.23172300	6.03531600	1.13856600
H	-2.98531400	4.68436300	-1.37040300
H	-2.05096400	6.35583400	0.21241900

7a

C	0.39433400	-1.62140500	-1.75551600
H	0.54618500	-1.42425500	-2.82017300
H	-0.08055100	-2.60332500	-1.63404600
N	1.67504000	-1.63059900	-1.06109000
C	2.69315500	-0.74219000	-1.62389200
O	1.36373100	-1.23238100	0.26837500
H	2.34579100	0.29640600	-1.73791300
H	2.85456300	-1.11999000	-2.64457200
H	-1.48630700	-0.95227700	-0.94916600
C	-0.47458900	-0.55445000	-1.08200000
C	0.18094700	-0.42096300	0.32496200
C	0.49262600	0.97122100	0.84620800
C	1.24454300	1.99799500	0.29139200
C	-0.07127500	1.12929600	2.10625800
C	1.39943700	3.17343600	1.02732300
H	1.69868300	1.90768400	-0.69270500
C	0.07685900	2.29009000	2.85012600
C	0.82439700	3.32355200	2.29203800
H	1.98375600	3.98859100	0.60624600
H	-0.37987700	2.37093400	3.83390000
H	0.96711400	4.25139000	2.84028900
N	-0.65937000	-0.95486800	1.39125900
C	-0.80081800	-0.10425700	2.46778300
O	-1.42509000	-0.34392300	3.48654500
C	-0.55789800	0.73987600	-1.88172000
O	0.27998400	1.00507200	-2.73973600
C	-1.06609300	-2.34748400	1.45492900
H	-0.24558600	-2.96352700	1.06447400
H	-1.17806300	-2.57925200	2.52193200
C	-1.76061100	2.79871300	-2.38700100
C	-2.66187000	1.36553300	-0.63024200
C	-1.95964900	3.97385300	-1.45262500
H	-2.64153200	2.69547000	-3.03976400
H	-0.87308100	2.92531600	-3.01176900
C	-2.84420400	2.61806300	0.21259700
H	-3.60149200	1.13631500	-1.15929800
H	-2.42988400	0.52212500	0.02576800
H	-2.17432300	4.88584000	-2.01865400
H	-1.04372900	4.13441900	-0.85456900
H	-3.71988600	2.51152700	0.86117600
H	-1.95438700	2.77192700	0.84997800
N	-1.60122900	1.57381900	-1.60981300
O	-3.06055800	3.75563700	-0.59339700
C	3.97889300	-0.79087100	-0.84826100
C	4.51606900	-2.00608100	-0.41748600
C	4.67592200	0.38647600	-0.58228800
C	5.72984100	-2.03960500	0.25929100
H	3.96437300	-2.92462100	-0.60762300
C	5.89592300	0.35630600	0.09057400
H	4.25661000	1.33897400	-0.90727500
C	6.42530400	-0.85829500	0.51371500
H	6.13588300	-2.99180800	0.59381300
H	6.42757600	1.28399900	0.29059500

H	7.37380800	-0.88600100	1.04525000
C	-2.35480900	-2.63748900	0.72750400
C	-2.42083200	-3.62967300	-0.25014000
C	-3.50770000	-1.90579300	1.02989100
C	-3.60968200	-3.87420500	-0.93564700
H	-1.53023400	-4.21573400	-0.47867400
C	-4.69368900	-2.14438100	0.34426400
H	-3.46591800	-1.14997900	1.81508900
C	-4.74625100	-3.12616700	-0.64511300
H	-3.64593500	-4.64861700	-1.69835800
H	-5.58459900	-1.56962100	0.58823200
H	-5.67425700	-3.31220500	-1.18049200

Crystallographic data

Table S1 Crystal data and structure refinement for compound **7a**.

Identification code	GF1501
Empirical formula	$C_{29}H_{29}N_3O_4$
Formula weight	483.55
Temperature/K	296(2)
Crystal system	orthorhombic
Space group	Pbca
a/Å	16.6179(4)
b/Å	12.1745(2)
c/Å	24.1593(5)
α/°	90
β/°	90
γ/°	90
Volume/Å^3	4887.78(17)
Z	8
ρ_{calc}g/cm^3	1.314
μ/mm^{-1}	0.088
F(000)	2048.0
Crystal size/mm^3	0.22 × 0.25 × 0.32
Radiation	MoKα (λ = 0.71073)
2Θ range for data collection/°	5.346 to 55.996
Index ranges	$-21 \le h \le 22$, $-15 \le k \le 16$, $-31 \le l \le 31$
Reflections collected	68416
Independent reflections	5881 [R_{int} = 0.0568, R_{sigma} = 0.0363]
Data/restraints/parameters	5881/0/326
Goodness-of-fit on F^2	1.011
Final R indexes [I>=2σ (I)]	R_1 = 0.0410, wR_2 = 0.0871
Final R indexes [all data]	R_1 = 0.0756, wR_2 = 0.1043
Largest diff. peak/hole / e Å^{-3}	0.24/-0.16

Table S2 Fractional atomic coordinates (×10^4) and equivalent isotropic displacement parameters (Å^2×10^3) for compound **7a**. U_{eq} is defined as 1/3 of of the trace of the orthogonalised U_{IJ} tensor.

**Atom**	***x***	***y***	***z***	**U(eq)**
O1	458.7(6)	7483.3(7)	4187.5(4)	33.8(2)
O2	2029.7(7)	10200.0(9)	4857.1(5)	51.4(3)
O3	929.5(7)	5589.5(9)	5548.3(4)	45.9(3)
O4	2867.6(9)	7959.0(11)	6482.6(5)	70.8(4)
N1	784.4(7)	6484.0(9)	3914.3(5)	30.7(3)
N2	1579.9(7)	8569.4(9)	4498.3(5)	30.6(3)
N3	1968.1(7)	6733.8(9)	5705.8(5)	35.7(3)
C4	1004.7(12)	3611.8(18)	2463.8(9)	69.1(6)
C5	1042.1(13)	4697.9(19)	2314.7(8)	71.1(6)
C6	756.9(11)	5500.8(15)	2667.3(7)	54.8(5)
C1	429.6(9)	5223.1(12)	3175.7(6)	37.1(3)
C7	122.7(9)	6103.6(12)	3558.4(6)	37.4(3)
C10	995.4(8)	7707.4(10)	4631.5(5)	27.9(3)
C18	2180.3(8)	8499.4(12)	4056.3(6)	34.3(3)
C19	1967.1(8)	9161.1(11)	3547.8(6)	32.9(3)
C20	1448.4(10)	8743.2(14)	3155.2(6)	45.5(4)
C21	1245.5(11)	9350.3(16)	2690.8(7)	55.5(5)
C22	1569.3(11)	10369.2(16)	2610.2(7)	56.4(5)
C3	684.1(12)	3324.3(15)	2966.1(8)	61.4(5)

C2	396.9(10)	4127.0(13)	3320.5(7)	45.9(4)
C17	1557.8(9)	9431.2(11)	4858.7(6)	34.6(3)
C12	880.2(9)	9218.5(11)	5241.0(6)	33.9(3)
C11	531.9(8)	8229.4(11)	5099.5(6)	31.1(3)
C16	-146.6(9)	7844.6(13)	5368.1(7)	42.7(4)
C15	-454.3(11)	8486.4(15)	5793.4(7)	52.3(4)
C14	-93.7(11)	9462.1(16)	5946.7(7)	55.0(5)
C13	576.5(10)	9849.5(13)	5670.6(7)	46.6(4)
C23	2087.4(11)	10794.3(15)	2995.2(8)	58.4(5)
C24	2284.7(10)	10195.5(13)	3462.0(7)	46.8(4)
C9	1407.4(8)	6575.8(10)	4761.9(5)	27.5(3)
C8	922.0(9)	5795.1(11)	4404.5(6)	32.2(3)
C25	1418.4(8)	6255.2(11)	5370.6(6)	30.5(3)
C26	2662.0(9)	7387.2(12)	5534.4(6)	40.4(4)
C27	2788.8(12)	8330.7(14)	5928.1(7)	57.0(5)
C28	2161.8(13)	7390.4(18)	6643.0(8)	70.3(6)
C29	2011.9(12)	6401.4(14)	6286.0(6)	52.5(4)

Table S3 Anisotropic displacement parameters (Å^2×10^3) for compound **7a**. The anisotropic displacement factor exponent takes the form: $-2\pi^2[h^2a^{*2}U_{11}+2hka^*b^*U_{12}+\ldots]$.

**Atom**	**U_{11}**	**U_{22}**	**U_{33}**	**U_{23}**	**U_{13}**	**U_{12}**
O1	37.5(5)	30.3(5)	33.7(5)	-4.1(4)	-7.2(4)	5.2(4)
O2	57.9(7)	37.9(6)	58.5(7)	-8.1(5)	-0.5(6)	-15.3(5)
O3	50.8(7)	47.8(6)	39.0(6)	11.4(5)	-3.0(5)	-15.9(5)
O4	92.7(10)	74.8(9)	45.0(7)	-7.4(6)	-25.6(7)	-24.3(8)
N1	35.3(6)	28.1(6)	28.7(6)	-4.6(5)	-2.3(5)	1.8(5)
N2	38.4(6)	26.1(6)	27.3(6)	0.3(5)	3.7(5)	-2.7(5)
N3	42.9(7)	36.9(6)	27.3(6)	-0.2(5)	-4.8(5)	-4.4(5)
C4	71.2(13)	75.5(14)	60.7(13)	-29.6(11)	2.3(11)	9.8(11)
C5	80.6(15)	90.9(16)	41.8(10)	-13.7(11)	16.9(10)	-8.3(12)
C6	69.0(12)	56.5(10)	38.8(9)	-3.7(8)	5.0(9)	-10.4(9)
C1	37.1(8)	43.7(8)	30.4(8)	-6.9(7)	-5.7(6)	-4.2(7)
C7	37.3(8)	42.0(8)	32.9(8)	-3.8(7)	-6.9(6)	-1.4(7)
C10	31.0(7)	26.4(6)	26.4(7)	1.4(5)	-0.6(6)	-1.3(5)
C18	32.9(7)	35.6(7)	34.5(8)	3.1(6)	4.2(6)	0.0(6)
C19	31.2(7)	37.2(8)	30.2(7)	1.0(6)	7.1(6)	1.3(6)
C20	53.8(10)	46.6(9)	35.9(8)	-0.9(7)	1.3(8)	-9.4(8)
C21	58.8(11)	73.8(12)	33.9(9)	0.5(9)	-7.5(8)	-3.6(10)
C22	61.8(12)	68.3(12)	39.1(9)	20.1(9)	1.4(9)	8.2(10)
C3	75.8(13)	49.2(10)	59.2(12)	-9.8(9)	-5.9(10)	9.1(9)
C2	53.8(10)	46.7(9)	37.1(9)	-3.9(7)	-2.7(8)	-0.2(8)
C17	42.1(8)	28.2(7)	33.6(8)	-0.3(6)	-4.5(7)	-0.1(6)
C12	41.7(8)	31.3(7)	28.7(7)	-0.5(6)	-2.2(6)	6.9(6)
C11	35.1(8)	30.4(7)	27.7(7)	2.2(6)	0.6(6)	7.3(6)
C16	41.0(9)	42.3(8)	44.8(9)	6.4(7)	8.5(7)	4.5(7)
C15	50(1)	61.5(11)	45.2(10)	10.5(9)	17.9(8)	15.9(9)
C14	65.3(12)	61.9(11)	37.7(9)	-5.2(8)	9.9(9)	24.8(10)
C13	59.5(11)	41.7(9)	38.6(9)	-9.0(7)	-2.8(8)	11.8(8)
C23	66.7(12)	50(1)	58.5(11)	20.2(9)	-0.6(10)	-8.0(9)
C24	50.1(10)	46.3(9)	44.0(9)	7.9(7)	-4.8(8)	-9.6(8)
C9	28.9(7)	26.2(6)	27.5(7)	-0.4(5)	-0.1(6)	-0.2(5)
C8	37.0(8)	28.0(7)	31.7(7)	-1.2(6)	-1.7(6)	-0.9(6)

C25	34.3(8)	26.4(6)	30.8(7)	0.8(6)	-1.4(6)	1.9(6)
C26	40.7(9)	40.8(8)	39.8(8)	1.0(7)	-8.2(7)	-7.6(7)
C27	70.7(12)	46.7(10)	53.5(11)	-2.3(8)	-20.8(9)	-12.1(9)
C28	88.7(15)	85.9(14)	36.4(10)	-12.4(10)	-4.7(10)	-12.2(12)
C29	65.6(12)	59.8(10)	31.9(8)	6.0(8)	-9.2(8)	-9.8(9)

Table S4 Bond lengths for compound **7a**.

Atom	Atom	Length/Å	Atom	Atom	Length/Å
O1	N1	1.4861(14)	C10	C9	1.5704(18)
O1	C10	1.4214(16)	C18	C19	1.5112(19)
O2	C17	1.2211(17)	C19	C20	1.379(2)
O3	C25	1.2252(16)	C19	C24	1.381(2)
O4	C27	1.420(2)	C20	C21	1.385(2)
O4	C28	1.416(2)	C21	C22	1.366(3)
N1	C7	1.4706(17)	C22	C23	1.369(3)
N1	C8	1.4691(17)	C3	C2	1.384(2)
N2	C10	1.4657(16)	C17	C12	1.479(2)
N2	C18	1.4638(17)	C12	C11	1.3790(19)
N2	C17	1.3640(17)	C12	C13	1.386(2)
N3	C25	1.3526(17)	C11	C16	1.383(2)
N3	C26	1.4609(18)	C16	C15	1.388(2)
N3	C29	1.4607(19)	C15	C14	1.381(3)
C4	C5	1.372(3)	C14	C13	1.381(2)
C4	C3	1.371(3)	C23	C24	1.382(2)
C5	C6	1.380(3)	C9	C8	1.5164(18)
C6	C1	1.385(2)	C9	C25	1.5217(19)
C1	C7	1.505(2)	C26	C27	1.506(2)
C1	C2	1.381(2)	C28	C29	1.502(3)
C10	C11	1.5086(18)			

Table S5 Bond angles for compound **7a**.

Atom	Atom	Atom	Angle/°	Atom	Atom	Atom	Angle/°
C10	O1	N1	105.29(9)	C21	C22	C23	119.61(16)
C28	O4	C27	109.73(14)	C4	C3	C2	120.10(18)
C7	N1	O1	104.19(10)	C1	C2	C3	120.79(16)
C8	N1	O1	99.56(9)	O2	C17	N2	124.78(14)
C8	N1	C7	114.07(11)	O2	C17	C12	128.68(13)
C18	N2	C10	124.76(11)	N2	C17	C12	106.52(12)
C17	N2	C10	113.13(11)	C11	C12	C17	108.52(12)
C17	N2	C18	121.93(11)	C11	C12	C13	121.11(14)
C25	N3	C26	126.72(12)	C13	C12	C17	130.35(14)
C25	N3	C29	119.26(12)	C12	C11	C10	109.84(12)
C29	N3	C26	112.55(12)	C12	C11	C16	121.45(13)
C3	C4	C5	119.75(18)	C16	C11	C10	128.71(13)
C4	C5	C6	120.32(18)	C11	C16	C15	117.19(15)
C5	C6	C1	120.61(17)	C14	C15	C16	121.52(16)
C6	C1	C7	120.27(14)	C15	C14	C13	120.93(15)
C2	C1	C6	118.43(15)	C14	C13	C12	117.77(16)
C2	C1	C7	121.30(14)	C22	C23	C24	120.27(16)
N1	C7	C1	109.28(12)	C19	C24	C23	120.85(16)

O1	C10	N2	112.81(10)	C8	C9	C10	101.75(10)
O1	C10	C11	109.03(11)	C8	C9	C25	113.32(11)
O1	C10	C9	104.87(10)	C25	C9	C10	115.10(11)
N2	C10	C11	101.61(10)	N1	C8	C9	100.60(10)
N2	C10	C9	112.54(11)	O3	C25	N3	121.53(13)
C11	C10	C9	116.22(11)	O3	C25	C9	120.02(12)
N2	C18	C19	113.71(11)	N3	C25	C9	118.44(12)
C20	C19	C18	120.60(13)	N3	C26	C27	110.28(14)
C20	C19	C24	118.16(14)	O4	C27	C26	111.44(13)
C24	C19	C18	121.24(13)	O4	C28	C29	111.85(16)
C19	C20	C21	120.84(15)	N3	C29	C28	109.71(14)
C22	C21	C20	120.26(16)				

Table S6 Hydrogen bonds for compound **7a**.

D	H	A	d(D-H)/Å	d(H-A)/Å	d(D-A)/Å	D-H-A/°
C7	H7B	O3[1]	0.97	2.56	3.4589(19)	153.4
C9	H9	O2[2]	0.98	2.45	3.0990(17)	123.3
C26	H26A	N2	0.97	2.55	3.4018(19)	146.1

[1]-X,1-Y,1-Z; [2]1/2-X,-1/2+Y,+Z

Table S7 Torsion angles for compound **7a**.

A	B	C	D	Angle/°	A	B	C	D	Angle/°
O1	N1	C7	C1	169.09(11)	C18	N2	C17	C12	-179.97(12)
O1	N1	C8	C9	-51.55(11)	C18	C19	C20	C21	179.42(14)
O1	C10	C11	C12	-124.79(12)	C18	C19	C24	C23	179.89(15)
O1	C10	C11	C16	54.67(18)	C19	C20	C21	C22	1.2(3)
O1	C10	C9	C8	-8.44(13)	C20	C19	C24	C23	0.0(2)
O1	C10	C9	C25	-131.39(11)	C20	C21	C22	C23	-0.9(3)
O2	C17	C12	C11	-177.64(15)	C21	C22	C23	C24	0.2(3)
O2	C17	C12	C13	4.1(3)	C22	C23	C24	C19	0.2(3)
O4	C28	C29	N3	-56.5(2)	C3	C4	C5	C6	-0.2(3)
N1	O1	C10	N2	99.39(11)	C2	C1	C7	N1	91.01(17)
N1	O1	C10	C11	-148.53(10)	C17	N2	C10	O1	122.89(12)
N1	O1	C10	C9	-23.42(12)	C17	N2	C10	C11	6.31(14)
N2	C10	C11	C12	-5.48(14)	C17	N2	C10	C9	-118.69(12)
N2	C10	C11	C16	173.97(14)	C17	N2	C18	C19	-81.87(16)
N2	C10	C9	C8	-131.42(11)	C17	C12	C11	C10	3.03(15)
N2	C10	C9	C25	105.63(13)	C17	C12	C11	C16	-176.47(13)
N2	C18	C19	C20	-82.16(17)	C17	C12	C13	C14	177.19(15)
N2	C18	C19	C24	97.95(16)	C12	C11	C16	C15	-1.2(2)
N2	C17	C12	C11	0.95(15)	C11	C10	C9	C8	112.02(13)
N2	C17	C12	C13	-177.35(15)	C11	C10	C9	C25	-10.94(16)
N3	C26	C27	O4	55.5(2)	C11	C12	C13	C14	-0.9(2)
C4	C5	C6	C1	0.0(3)	C11	C16	C15	C14	-0.7(2)
C4	C3	C2	C1	-0.1(3)	C16	C15	C14	C13	1.8(3)
C5	C4	C3	C2	0.2(3)	C15	C14	C13	C12	-0.9(2)
C5	C6	C1	C7	179.79(16)	C13	C12	C11	C10	-178.48(13)
C5	C6	C1	C2	0.1(3)	C13	C12	C11	C16	2.0(2)
C6	C1	C7	N1	-88.70(17)	C24	C19	C20	C21	-0.7(2)
C6	C1	C2	C3	0.0(2)	C9	C10	C11	C12	117.02(13)
C7	N1	C8	C9	-161.89(11)	C9	C10	C11	C16	-63.52(19)

C7	C1	C2	C3	-179.72(15)	C8	N1	C7	C1	-83.41(14)
C10	O1	N1	C7	165.69(10)	C8	C9	C25	O3	-15.57(18)
C10	O1	N1	C8	47.70(11)	C8	C9	C25	N3	165.44(12)
C10	N2	C18	C19	103.50(14)	C25	N3	C26	C27	142.16(14)
C10	N2	C17	O2	173.88(13)	C25	N3	C29	C28	-140.79(15)
C10	N2	C17	C12	-4.77(15)	C25	C9	C8	N1	161.22(11)
C10	C11	C16	C15	179.45(14)	C26	N3	C25	O3	168.82(14)
C10	C9	C8	N1	37.06(12)	C26	N3	C25	C9	-12.2(2)
C10	C9	C25	O3	100.98(14)	C26	N3	C29	C28	52.11(19)
C10	C9	C25	N3	-78.01(15)	C27	O4	C28	C29	60.7(2)
C18	N2	C10	O1	-62.07(16)	C28	O4	C27	C26	-59.9(2)
C18	N2	C10	C11	-178.66(12)	C29	N3	C25	O3	3.7(2)
C18	N2	C10	C9	56.35(16)	C29	N3	C25	C9	-177.31(13)
C18	N2	C17	O2	-1.3(2)	C29	N3	C26	C27	-51.89(17)

Table S8 Hydrogen atom coordinates (Å×10^4) and isotropic displacement parameters (Å^2×10^3) for compound **7a**.

**Atom**	***x***	***y***	***z***	**U(eq)**
H4	1196	3073	2225	83
H5	1261	4894	1974	85
H6	785	6235	2563	66
H7A	-87	6714	3344	45
H7B	-310	5814	3785	45
H18A	2244	7736	3951	41
H18B	2693	8755	4198	41
H20	1232	8045	3203	55
H21	887	9063	2433	67
H22	1439	10772	2296	68
H3	660	2589	3069	74
H2	179	3926	3660	55
H16	-387	7184	5268	51
H15	-914	8254	5979	63
H14	-305	9864	6240	66
H13	816	10511	5769	56
H23	2308	11489	2942	70
H24	2636	10493	3722	56
H9	1962	6594	4624	33
H8A	1225	5139	4314	39
H8B	420	5589	4581	39
H26A	2575	7668	5164	48
H26B	3139	6928	5528	48
H27A	3270	8729	5823	68
H27B	2336	8831	5903	68
H28A	1704	7883	6618	84
H28B	2213	7160	7026	84
H29A	2444	5874	6335	63
H29B	1511	6053	6394	63
